# Strategic treatment optimization for HCV (STOPHCV1): a randomised controlled trial of ultrashort duration therapy for chronic hepatitis C

**DOI:** 10.12688/wellcomeopenres.16594.1

**Published:** 2021-04-29

**Authors:** Graham S. Cooke, Sarah Pett, Leanne McCabe, Chris Jones, Richard Gilson, Sumita Verma, Stephen D. Ryder, Jane D. Collier, Stephen T. Barclay, Aftab Ala, Sanjay Bhagani, Mark Nelson, Chinlye Ch'Ng, Ben Stone, Martin Wiselka, Daniel Forton, Stuart McPherson, Rachel Halford, Dung Nguyen, David Smith, Azim Ansari, Emily Dennis, Fleur Hudson, Eleanor J. Barnes, Ann Sarah Walker

**Affiliations:** 1 Department of Infectious Disease, Imperial College London, London, W2 1NY, UK; 2 NIHR Biomedical Research Centre, Imperial College NHS Trust, London, W2 1NY, UK; 3 MRC Clinical Trials Unit, University College London Medical School, London, UK; 4 Mortimer Market Centre, Central and North West London NHS Foundation Trust, London, UK; 5 Institute of Global Health, University College London Medical School, London, UK; 6 Hepatology, Brighton and Sussex Medical School, Brighton, UK; 7 Hepatology, Nottingham University Hospitals NHS Trust, Nottingham, UK; 8 Hepatology, John Radcliffe Hospital, Oxford, UK; 9 Gastroenterology, Glasgow Royal Infirmary, Glasgow, UK; 10 Clinical and Experimental Medicine, University of Surrey, Guilford, UK; 11 Infectious Diseases, Royal Free Hampstead NHS Trust Hospital, London, UK; 12 HIV Medicine, Chelsea & Westminster NHS Trust, London, UK; 13 Swansea Bay University Health Board, Swansea, UK; 14 Infectious Diseases, Sheffield Teaching Hospitals Nhs Foundation Trust, Sheffield, UK; 15 Infectious Diseases, University Hospitals of Leicester NHS Trust, Leicester, UK; 16 Hepatology, St George's Hospital, London, London, UK; 17 Heaptology, Newcastle Upon Tyne Hospitals NHS Trust, Newcastle, UK; 18 Hepatitis C Trust, London, UK; 19 Peter Medawar Buildling for Pathogen Research, Oxford, UK; 20 Translational Gastroenterology Unit, John Radcliffe Hospital, Oxford, UK

**Keywords:** hepatitis, clinical trial, short course, treatment, ribavirin

## Abstract

**Background: **The world health organization (WHO) has identified the need for a better understanding of which patients with hepatitis C virus (HCV) can be cured with ultrashort course HCV therapy.

**Methods: **A total of 202 individuals with chronic HCV were randomised to fixed-duration shortened therapy (8 weeks) vs variable-duration ultrashort strategies (VUS1/2). Participants not cured following first-line treatment were retreated with 12 weeks’ sofosbuvir/ledipasvir/ribavirin. The primary outcome was sustained virological response 12 weeks (SVR12) after first-line treatment and retreatment. Participants were factorially randomised to receive ribavirin with first-line treatment.

**Results: **All evaluable participants achieved SVR12 overall (197/197, 100% [95% CI 98-100]) demonstrating non-inferiority between fixed-duration and variable-duration strategies (difference 0% [95% CI -3.8%, +3.7%], 4% pre-specified non-inferiority margin). First-line SVR12 was 91% [86%-97%] (92/101) for fixed-duration vs 48% [39%-57%] (47/98) for variable-duration, but was significantly higher for VUS2 (72% [56%-87%] (23/32)) than VUS1 (36% [25%-48%] (24/66)). Overall, first-line SVR12 was 72% [65%-78%] (70/101) without ribavirin and 68% [61%-76%] (69/98) with ribavirin (p=0.48). At treatment failure, the emergence of viral resistance was lower with ribavirin (12% [2%-30%] (3/26)) than without (38% [21%-58%] (11/29), p=0.01).

**Conclusions: **Unsuccessful first-line short-course therapy did not compromise retreatment with sofosbuvir/ledipasvir/ribavirin (100% SVR12). SVR12 rates were significantly increased when ultrashort treatment varied between 4-7 weeks rather than 4-6 weeks. Ribavirin significantly reduced resistance emergence in those failing first-line therapy.

**ISRCTN Registration**: 37915093 (11/04/2016).

## Introduction

The recent and rapid development of treatment for hepatitis C virus (HCV) has enabled an ambitious strategy for the elimination of viral hepatitis as a global public health threat by 2030, with the target of treating 80% of those chronically infected with HCV ^1^. Although licensed durations of 8–12 weeks’ therapy with directly acting antivirals (DAAs) are significantly shorter, more tolerable and more effective than previous interferon-based therapies ^2^, there are patients who still find it challenging to complete a full treatment course. Such patients will become an increasingly important part of clinical practice as treatment coverage expands to reach marginalised groups, and world health organization (WHO) treatment guidelines highlight the need to understand the factors that could be used to select patients for successful short course treatment ^3^.

Shorter treatment courses of licensed therapies are likely to improve adherence, including in those with active illicit drug use ^4, 5^. In acute or recent HCV infection, shortened courses of licensed therapy may have sufficiently high efficacy to be recommended routinely ^6^. However, in chronic infection there is limited data to select patients able to achieve high cure rates with short duration therapy. Unselected short duration treatment has been able to achieve cure rates of 20–40% with 4 weeks and 57–95% with 6 weeks treatment in small Phase II studies ^7– 9^, but few of these combinations or durations were subsequently licensed for use. For licensed therapies, baseline viral load (<6,000,000 IU/mL) ^10^ and subgenotype ^11^ have been recommended to shorten therapy from 12 to 8 weeks, but there are no validated criteria to recommend less than 8 weeks therapy in chronic infection.

For a clinician deciding whether to start treatment in a patient considered at high risk of not completing therapy, there is a potential concern that emerging resistance with virological failure may compromise future treatment options. However, it is also possible that with shorter courses of treatment, less resistance may emerge. *In vitro* evidence suggests the addition of ribavirin, a generically available guanosine analogue, may improve rates of virological cure with shorter treatment courses ^12^ and could reduce the emergence of resistance in those failing treatment when added to short-course therapy ^13^. However, these hypotheses have not been tested in a randomised trial.

We performed a strategic post-licensing randomised controlled trial in HCV-infected participants with mild liver disease to evaluate strategies for short-course treatment, the impact of treatment failure on retreatment and the role of adjunctive ribavirin in short-course therapy.

## Methods

### Design

**Figure 1.**
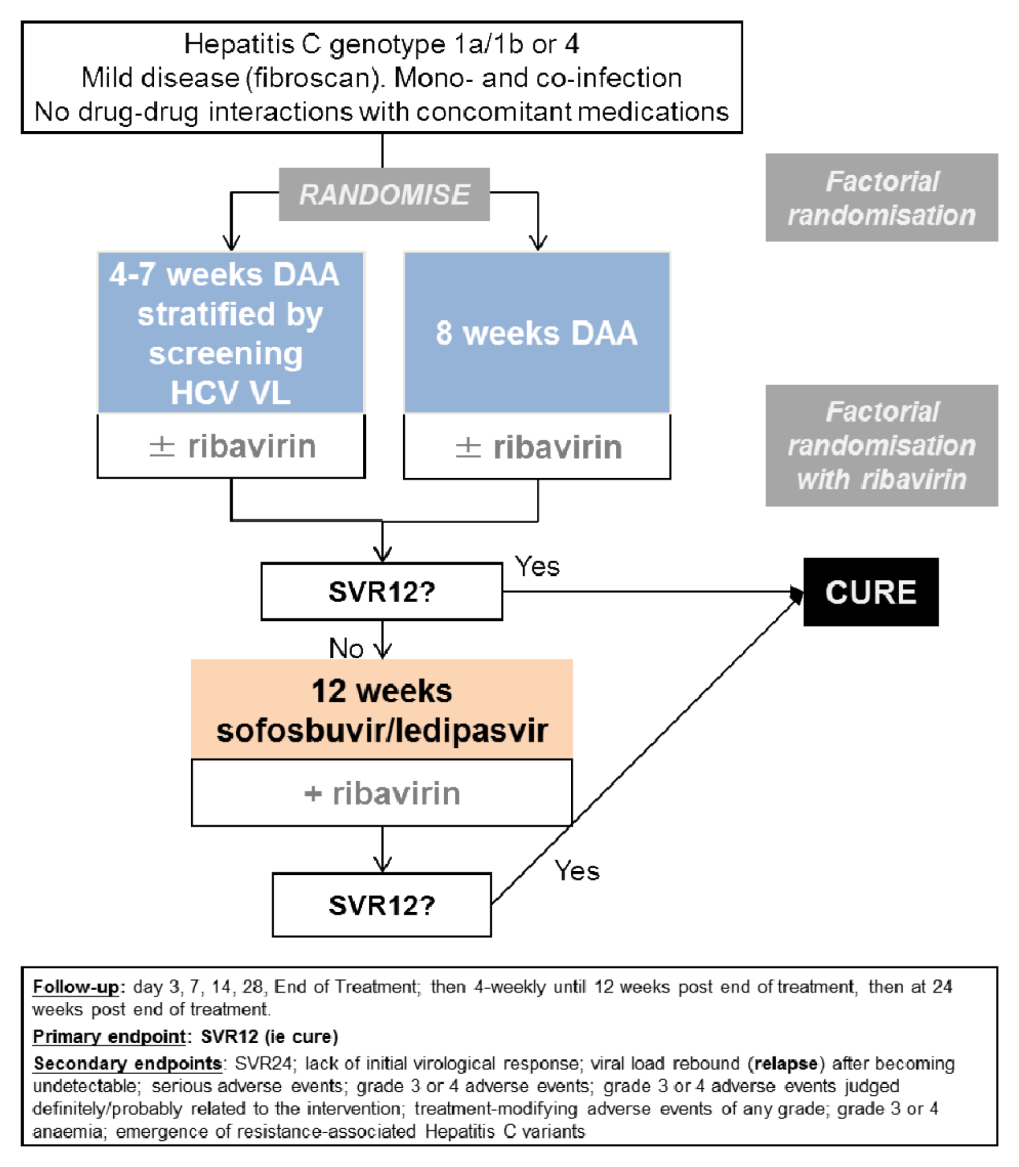
Trial schematic. Note: DAA: direct-acting antivirals. The ribavirin randomisation was a partial factorial in those randomised to a shorter course than the full licensed duration of therapy (the vast majority of participants recruited to the trial).

We conducted a multi-centre, randomised, open-label, factorial, parallel group non-inferiority trial of adults with mild chronic HCV in 14 UK centres ( Figure 1). The trial was approved by the Cambridgeshire South Research Ethics Committee (15/EE/0435). The trial was registered at ISRCTN (37915093, 11 ^th^ April 2016), and EudraCT (2015-005004-28, 31 ^st^ December 2015). This study is reported in line with the Consolidated Standards of Reporting Trials (CONSORT) ^14^.

### Participants

Eligible participants were adults (≥18 years) infected with HCV genotype 1a/1b/4 for ≥6 months, with consistently detectable viremia 6 months before randomisation, no evidence of significant liver fibrosis (Fibroscan score ≤7.1kPa equivalent to F0-F1 ^15^), body mass index (BMI) ≥18kg/m ^2^, HCV viral load (VL) detectable but <10 million IU/ml at screening, no previous DAA exposure for current infection (previous pegylated-interferon/ribavirin allowed) and with laboratory results meeting minimum thresholds (platelets ≥60×10 ^9^/L, haemoglobin >12g/dL (male) or >11g/dL (female), creatinine clearance (estimated using Cockcroft-Gault) ≥60ml/min, international normalised ratio (INR) <1.5). Individuals co-infected with HIV were eligible if HIV VL had been <50 copies/ml for >24 weeks on anti-HIV drugs. Participants were excluded if they had a history of malignancy within 5 years, any history of pre-existing cardiac disease or haemoglobinopathies, or a current disorder which may cause ongoing liver disease, may negatively impact the participant’s ability to adhere to the study or might limit the participant’s life expectancy. Participants were also excluded if they were hypersensitive to any of the study drugs, currently taking any medication known to interact with study medication or had used other investigational products within 60 days of screening. Female participants were excluded if lactating, pregnant, planning to become pregnant or not willing to use effective contraception (excluding products containing ethinyl-oestradiol) including up to four months after the study. Male participants were excluded if they were planning pregnancy with a partner or not willing to use effective contraception for up to seven months after the study. Participants gave written informed consent after explanation of the aims, methods, benefits and potential hazards of the trial and before any trial-specific procedures were performed/any blood taken for the trial.

### Trial setting

Participants were recruited from 14 UK NHS Hospital Trusts: Singleton Hospital, Swansea Bay University Health Board; University Hospitals of Leicester NHS Trust; Imperial College NHS Trust; St George’s Healthcare NHS Foundation Trust; Royal Free Hospital NHS Foundation Trust; Nottingham University Hospitals NHS Trust; Royal Surrey County Hospital NHS Foundation Trust; Brighton and Sussex University Hospitals NHS Trust; John Radcliffe Hospital Oxford University Hospitals NHS Foundation Trust; Glasgow Royal Infirmary NHS Greater Glasgow and Clyde; Newcastle Freeman Hospital Newcastle Upon Tyne Hospitals NHS Foundation Trust; Chelsea and Westminster Hospital NHS Foundation Trust; Central and North West London NHS Foundation Trust; and Sheffield Teaching Hospitals NHS Foundation Trust.

The main criteria for selecting participating hospitals was that they had the potential for recruiting the required number of chronic (>6 months) HCV genotype 1a/1b/4 infected participants within the agreed recruitment period. This was established by the use of a trial specific site survey. Site also needed to meet the following criteria: no competing studies that would impact on the ability to enrol quickly to the trial; turnaround of no more than 7 days for HCV viral load test results; ability to provide 24 hour cover for trial patients; local governance approval likely to take <3 months.

### Randomisation

Participants were randomised 1:1 to variable ultrashort-course treatment strategy (VUS) or fixed 56 days of first-line treatment. Individuals were also randomised 1:1 using a factorial design to adjunctive ribavirin or no ribavirin with first-line therapy. Randomisation determined duration of first-line therapy rather than choice of DAAs which was pre-specified by the investigator before randomisation based on local availability from (i) [genotype 1a/1b] co-formulated ombitasvir/paritaprevir/ritonavir once daily plus separate dasabuvir once-daily (total 25mg/150mg/100 mg plus 500mg, respectively) (ii) [genotype 4] ombitasvir/paritaprevir/ritonavir 25/150/100mg once-daily (iii) [genotype 1a/1b/4] glecaprevir/pibrentasvir 300/120mg once-daily (only available after 1 November 2017). Ribavirin dosing was weight-based twice-daily (<75kg 1000mg/day, ≥75kg 1200mg/day). All drugs were taken orally.

### Intervention and procedures

**Figure 2.**
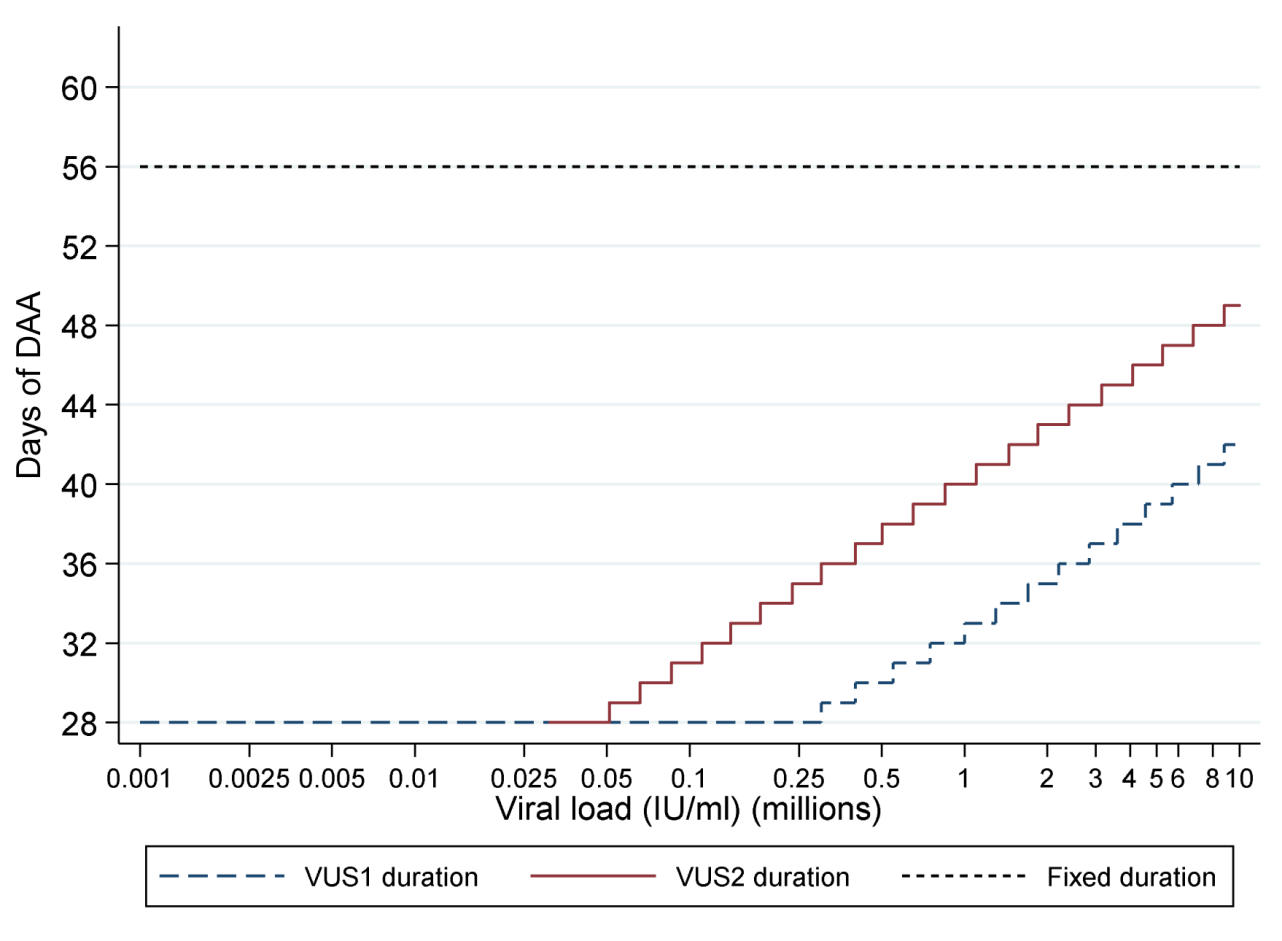
Duration of first-line treatment in the variable-duration group. DAA: direct acting antivirals; VUS1: variable ultra-short 1 (4–6 weeks); VUS2: variable ultra-short (4–7 weeks). Note: Lines represent protocol determined treatment duration according to screening viral load (x axis).

For participants allocated to VUS, the duration of first-line therapy varied between 28 and 42 (mean 32; before 1 April 2017) or 49 (mean 39; after 1 April 2017) days determined by the baseline screening VL using a continuous scale ( Table 1, Table 2, Figure 2). Blinding was not used because the primary end-point was an objective measure of viraemia blinded to clinical data measured in routine laboratories without knowledge of randomisation.

**Table 1.** Duration of first-line treatment in the variable-duration group by protocol version.

From HCV VL (IU/ml)	To HCV VL (IU/ml)	Days if randomised before 01/04/2017 (VUS1)	Days if randomised after 01/04/2017 (VUS2)
LLOQ	50,000	28	28
50,001	65,000	28	29
65,001	82,500	28	30
82,501	110,000	28	31
100,001	140,000	28	32
150,001	180,000	28	33
175,001	235,000	28	34
225,001	300,000	28	35
300,001	400,000	29	36
400,001	500,000	30	37
500,001	550,000	30	38
550,001	650,000	31	38
650,001	750,000	31	39
750,001	850,000	32	39
850,001	1,100,000	32	40
1,100,001	1,300,000	33	41
1,300,001	1,450,000	34	41
1,450,001	1,700,000	34	42
1,700,001	1,850,000	35	42
1,850,001	2,200,000	35	43
2,200,001	2,400,000	36	43
2,400,001	2,850,000	36	44
2,850,001	3,150,000	37	44
3,150,001	3,600,000	37	45
3,600,001	4,100,000	38	45
4,050,001	4,550,000	38	46
4,550,001	5,250,000	39	46
5,250,001	5,700,000	39	47
5,700,001	6,800,000	40	47
6,800,001	7,100,000	40	48
7,100,001	8,800,000	41	48
8,800,001	upwards	42	49

Note: VUS: variable ultra-short

**Table 2.** Summary of length of treatment received in the variable duration arm.

	VUS1 N=68	VUS2 N=34	Total N=102
4–5 weeks (28–34 days)	48 (71%)	11 (32%)	59 (58%)
5–6 weeks (35–42 days)	18 (26%)	13 (38%)	31 (30%)
6–7 weeks (42–49 days)	2 (3%)	10 (29%)	12 (12%)
Mean (SD)	32 (4.2)	39 (5.6)	35 (5.7)

All patients failing treatment were retreated as soon as practicable with 12 weeks of oral sofosbuvir 400mg/ledipasvir 90mg once-daily and weight-based oral ribavirin twice-daily (dosing as above). Treatment failure was defined as (i) two consecutive measurements of HCV VL above the lower limit of quantification (LLOQ) (taken at least one week apart) after two consecutive visits with HCV VL <LLOQ at any time, with the latter confirmatory measurement also being >2000 IU/mL, or (ii) two consecutive measurements of HCV VL (taken at least one week apart) that were >1log _10_ increase above the nadir on treatment and >2000 IU/mL at any time.

All participants were followed by the site teams for 24 weeks after the end of first-line treatment or re-treatment (where applicable) for evaluation of virological response and toxicity. Participants on first-line therapy had clinical assessments on days 3, 7, 10, 14, 28 and end of treatment (EOT, where EOT was not day-28) followed by weeks 2, 4, 8, 12 and 24 after EOT. All outcome measures were assessed at these clinic visits.

### Randomisation

Randomisation was performed via a computer-generated programme at the STOP-HCV-1 Co-ordinating Centre (MRC CTU at UCL). Patients were allocated 1:1 using a factorial design to each of: biomarker-stratified variable ultrashort vs fixed duration; adjunctive ribavirin or not (a partial factorial in those randomised to a shorter course than the full licensed duration of therapy). Randomisation was stratified by study centre, HCV genotype and study drug regimen using a minimisation algorithm incorporating a probabilistic element incorporated securely into the online trial database. Randomisation determined the duration of first-line therapy rather than the choice of DAAs which was pre-specified by the investigator before randomisation based on local availability.

Each allocation was generated within the trial database only at the point of randomisation after it was confirmed the participant was eligible and was to be randomised. Allocations were generated using minimisation with a probabilistic element, so there was no pre-determined allocation sequence to conceal. To further conceal the potential allocation, study centres were not informed of the randomisation strata.

On the day of randomisation, participant eligibility was checked at sites and the data confirming eligibility was entered onto a case record form and sent to MRC CTU. The data was entered onto the database at MRC CTU and checked for eligibility again. Once confirmed that the participant was eligible, the database would perform randomisation using the computer-generated programme. Sites were then informed of the allocation and length of DAA treatment required for the participant.

### Sample size

*A priori* power calculation assumed 88% of participants would achieve SVR12 on first-line fixed-duration, and that SVR12 would be 85% on retreatment (significantly lower than the actual retreatment success rate, below), leading to an overall cure rate of 98% (first-line plus retreatment) in the control group. Assuming 98% cure rate in the fixed-duration group, 80% power, one-sided alpha 0.025, and a 5% loss to follow-up, 408 participants were needed to demonstrate non-inferiority with a 4% margin. The choice of the 4% margin was based on clinical judgement and to ensure that overall cure rates in the variable-duration group would be well over 90% if non-inferiority was demonstrated. Interim data were reviewed by an independent Data Monitoring Committee (DMC) (4 biannual meetings). The protocol stipulated that the DMC could alter the first-line treatment strategy if there was strong evidence the SVR12 rate for VUS1 was less than 65%. After the DMC meeting in April 2017, the DAA duration strategy was changed from 4–6 weeks (VUS1) to 4–7 weeks (VUS2) ( Table 1). All participants randomised from the 1st April 2017 were treated under VUS2. The trial closed in August 2018 when no further recruitment was possible. By this time, the great majority of patients with viraemia were unable to engage with treatment *per se*, and not suitable for inclusion in this study.

### Outcomes

The primary outcome was sustained virological response 12 (SVR12, plasma HCV VL <LLOQ without prior failure 12 weeks after the end of the combined first and any re-treatment phases). For ribavirin comparison the primary outcome was SVR12 after first-line treatment only. Secondary outcomes were SVR12 after first-line treatment (where not the primary outcome), SVR12 after the end of the combined first and any re-treatment phases (where not the primary outcome), SVR24 (24 weeks) after the end of the combined first and any re-treatment phases, SVR24 after first-line treatment only, lack of initial virological response, viral load rebound after becoming undetectable, serious adverse events, grade 3/4 adverse events, grade 3/4 adverse events judged definitely/probably related to interventions, treatment-modifying adverse events (any grade), grade 3/4 anaemia and emergence of resistance-associated HCV variants. Adverse events were graded following the Division of AIDS grading tables. Full genome sequence was obtained by RNA Sequencing on Illumina Miseq platform following target enrichment with a library of genotype specific HCV capture probes as described previously ^16^.

### Statistical analysis

Randomised groups were compared following the principle of intention-to-treat (including all follow-up regardless of changes to treatment) using binomial regression (risk difference scale) to estimate risk differences for binary outcomes, Kaplan-Meier and Cox regression for time to first-line failure (with competing risks methods for its components, primary failure and VL rebound), and generalised estimating equations with independent working correlation for global tests of repeated measures (adjusted for baseline for continuous measures). A per-protocol analysis included patients receiving >90% and <100% of the prescribed duration of first-line treatment and where the difference between screening and enrolment HCV RNA values would have led to a difference of ≤2 days in allocated duration of DAAs had they been allocated to the variable-duration group. Primary analyses of outcomes restricted to first-line therapy were stratified by first-line DAA strategy in place (VUS1 (before 1 April 2017) or VUS2 (after 1 April 2017)) as a main effect, and as an interaction with randomised group (fixed-duration vs variable-duration), where the p-value for the interaction term was <0.05. Primary analyses of outcomes including retreatment were unstratified, reflecting the overall strategy comparison and because no patients failed after receiving retreatment. Analyses used Stata v15.1. No adjustment was made for multiple testing. All subgroups within the subgroup analyses were pre-specified in the protocol. Further information on statistical methods and results can be found as extended data ^17^.

## Results

**Figure 3.**
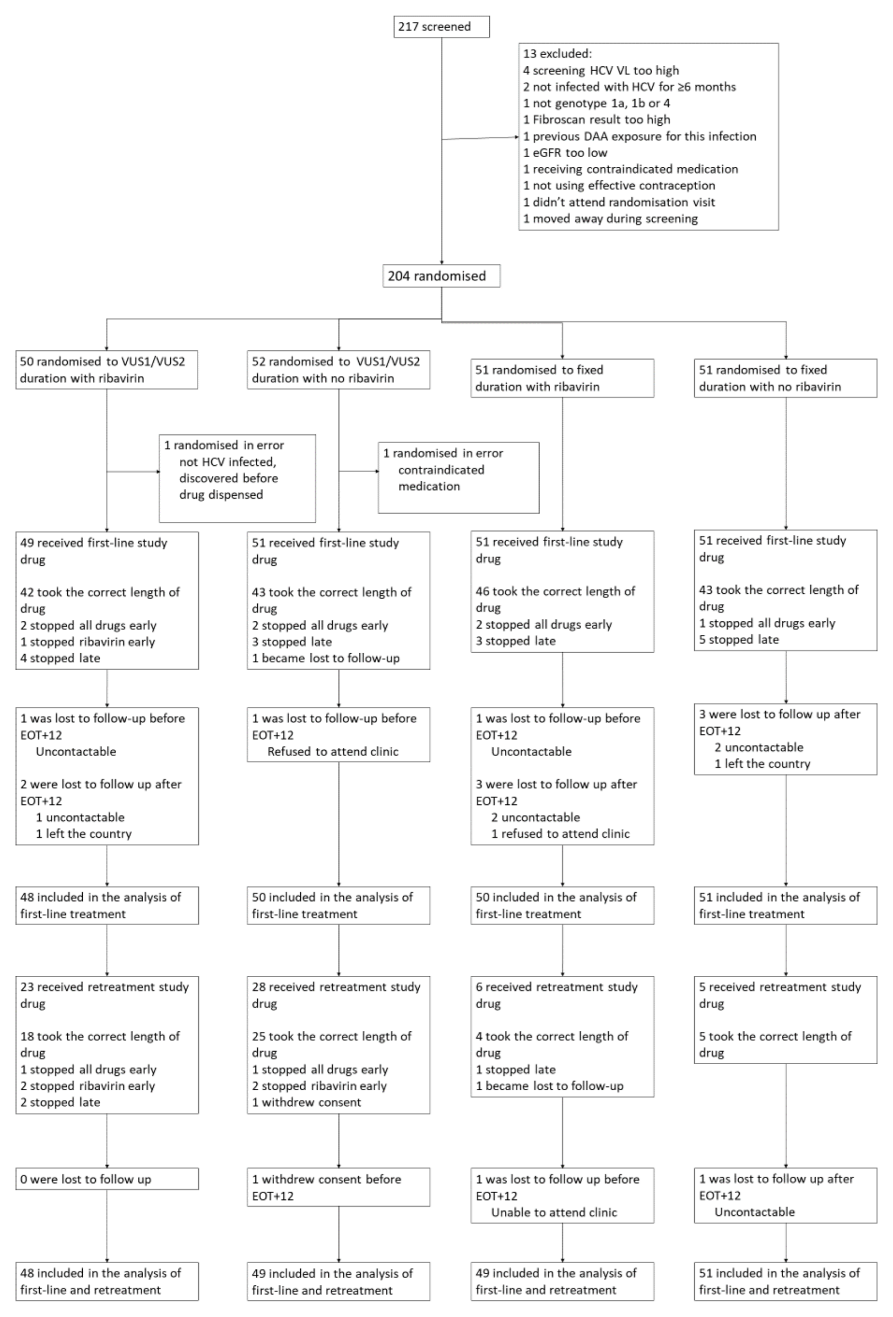
Trial profile. Note: EOT+12: 12 weeks after the end of treatment; EOT+24 24 weeks after the end of treatment. Some patients lost to follow up are included in the analysis based on local VLs from clinics.

Between 18 March 2016 and 28 August 2018, 204 participants from 14 UK centres were randomised ( Figure 3). Two participants were randomised in error and excluded, leaving 202 (102 fixed-duration, 100 variable-duration; 100 ribavirin, 102 no-ribavirin) participants in the analyses ^18^.

**Figure 4.**
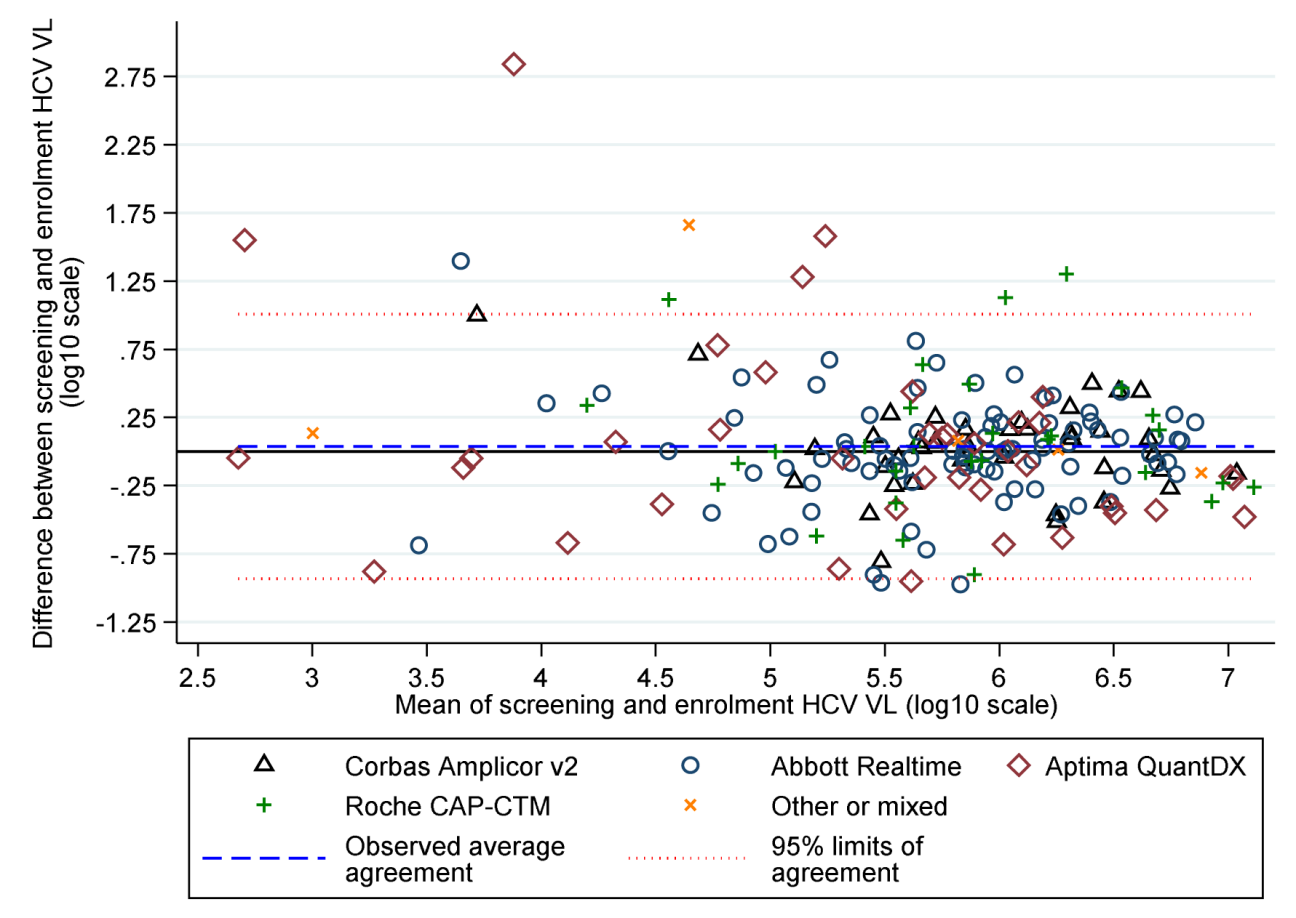
HCV VL at screening and enrolment by assay.

Baseline characteristics were broadly balanced between randomised groups ( Table 3) and across strategies (before vs after 1 April 2017). Median screening and enrolment HCV VL were 711423 and 741946 IU/mL in samples taken a median (IQR) 19 (13,33) days apart. Whilst the median (IQR) difference was 0.01 (-0.19,+0.21) log _10_ IU/mL, absolute differences were greater ( Figure 4), leading to 57 (28%) participants being excluded from the per-protocol analysis because they would have received a difference of ≥3 days of DAAs had this been determined by the enrolment rather than the screening VL (31 (23%) VUS1 vs 26 (39%) VUS2, because the second strategy received more drug overall).

**Table 3.** Characteristics at randomisation.

	Total (N=202)	VUS duration (N=100)	Fixed duration (N=102)	Ribavirin (N=100)	No ribavirin (N=102)
Randomised under first protocol (VUS1)	136 (67%)	68 (68%)	68 (67%)	68 (68%)	68 (67%)
Age (years)	45.5 (37.5, 53.0)	45.2 (38.8, 51.6)	46.3 (36.6, 54.1)	46.1 (36.7, 52.4)	44.8 (37.7, 54.1)
Female at birth	62 (31%)	28 (28%)	34 (33%)	34 (34%)	28 (27%)
BMI (kg/m ^2^)	24.9 (22.2, 27.2)	24.9 (22.6, 26.7)	24.9 (21.8, 27.7)	23.7 (21.7, 26.5)	25.8 (23.3, 27.6)
White ethnicity	176 (87%)	89 (89%)	87 (85%)	89 (89%)	87 (85%)
Weight (kg)	74.0 (66.0, 84.6)	73.0 (65.9, 84.1)	76.1 (66.0, 85.9)	69.9 (63.8, 82.3)	78.9 (68.5, 86.8)
Screening HCV viral load (IU/ml)	711423 (218776,1995262)	790664 (214388, 1917731)	687916 (220000, 2381846)	700272 (169717, 2071064)	750523 (275000, 1949844)
Enrolment HCV viral load (IU/ml) n=199	741946 (249097,1872136)	801000 (251188, 1500000)	(614047 (248000, 2238721)	657858 (178842, 1500000)	801000 (385595, 2200000)
HCV genotype/subgenotype: 1a	166 (82%)	82 (82%)	84 (82%)	84 (84%)	82 (80%)
1b	34 (17%)	17 (17%)	17 (17%)	16 (16%)	18 (18%)
4	2 (1%)	1 (1%)	1 (1%)	0	2 (2%)
HIV coinfected	68 (34%)	32 (32%)	36 (35%)	35 (35%)	33 (32%)
Fibroscan result (kPa)	4.9 (4.2, 5.8)	5.0 (4.3, 5.9)	4.8 (4.1, 5.5)	4.8 (4.4, 5.8)	4.9 (4.1, 5.9)
Haemoglobin (g/dl)	14.7 (14.0, 15.6)	14.8 (14.1, 15.6)	14.7 (13.8, 15.6)	14.7 (13.8 , 15.6)	14.8 (14.0, 15.7)
ALT (IU/ml)	52 (34, 87)	50 (34, 90)	54 (34, 87)	51 (35, 89)	54 (31, 87)
AST (IU/l) n=189	38 (30, 57)	38 (29, 57)	38 (31, 58)	39 (31, 55)	38 (29, 58)
ALP (IU/l)	72 (59, 91)	71 (59, 87)	75 (59, 94)	76 (61, 95)	69 (58, 85)
eGFR (ml/min)	109 (93, 131)	109 (94, 126)	109 (92, 138)	107 (92, 126)	110 (93, 133)
Total bilirubin (umol/l)	9 ( 6, 12)	8 (6, 11)	9 (6, 12)	9 (6, 12)	9 (6, 12)
IL28b genotype *: CC	60 (30%)	32 (32%)	28 (27%)	29 (29%)	31 (30%)
CT	106 (52%)	51 (51%)	55 (54%)	56 (56%)	50 (49%)
TT	27 (13%)	14 (14%)	13 (13%)	11 (11%)	16 (16%)
No result	9 (4%)	3 (3%)	6 (6%)	4 (4%)	5 (5%)
Previously unsuccessfully treated with interferon and/or ribavirin	24 (12%)	12 (12%)	12 (12%)	11 (11%)	13 (13%)
Ever spontaneously cleared and re- infected	6 (3%)	4 (4%)	2 (2%)	2 (2%)	4 (4%)
Ever successfully treated with interferon and/or ribavirin and re-infected	10 (5%)	5 (5%)	5 (5%)	5 (5%)	5 (5%)
Current/recent alcoholism/alcohol abuse	13 (6%)	5 (5%)	8 (8%)	7 (7%)	6 (6%)
Current/recent illicit substance abuse	64 (32%)	31 (31%)	33 (32%)	28 (28%)	26 (25%)
Treated with paritaprevir\ombitasvir\ dasabuvir	198 (98%)	98 (98%)	100 (98%)	100 (100%)	98 (96%)
Treated with paritaprevir\ombitasvir	2 (1%)	1 (1%)	1 (1%)	0	2 (2%)
Treated with glecaprevir\pibrentasvir	2 (1%)	1 (1%)	1 (1%)	0	2 (2%)

* Result from whole genome sequencing or from Epistem point of care test if genotyping result not available.

Note: showing n (%) for categorical factors, or median (IQR) for continuous factors. Missing data indicated by denominators in the row label. As an indicator of imbalance, P>0.05 for all comparisons of baseline characteristics between groups other than BMI (p<0.001), weight (p=0.003) and ALP (p=0.04) between ribavirin and no ribavirin groups.

### Follow-up and treatment received

**Figure 5.**
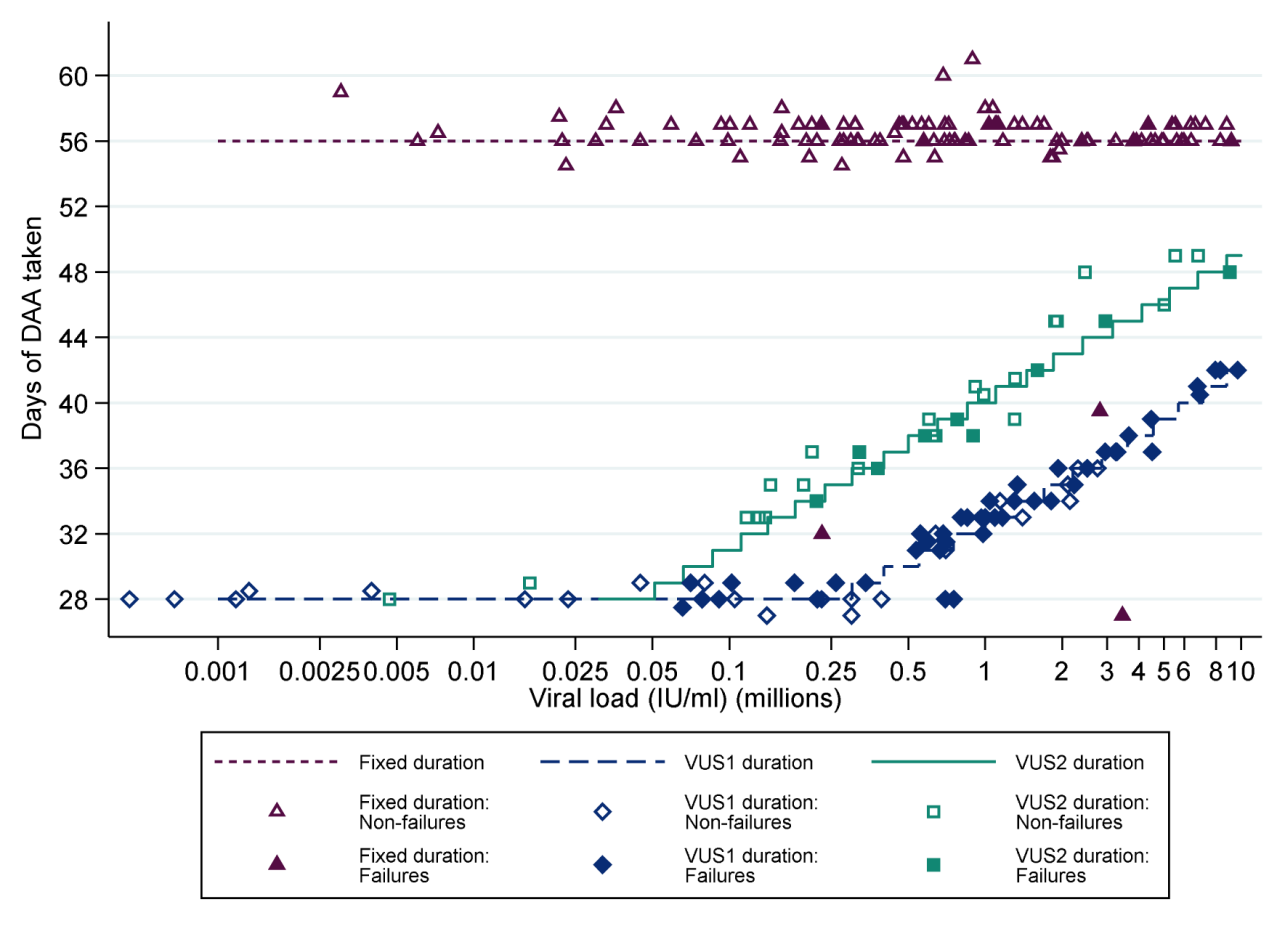
Self-reported treatment duration and first-line SVR24. Note: Lines represent protocol determined treatment duration according to screening viral load (x axis). Symbols represent self-reported individual duration of therapy. Patients could stop DAAs early for adverse events or personal reasons, and take DAAs for longer than prescribed if any missed doses were taken at the end of treatment. Excludes one patient lost-to-follow-up during first-line whose outcome on first-line is unknown.

**Figure 6.**
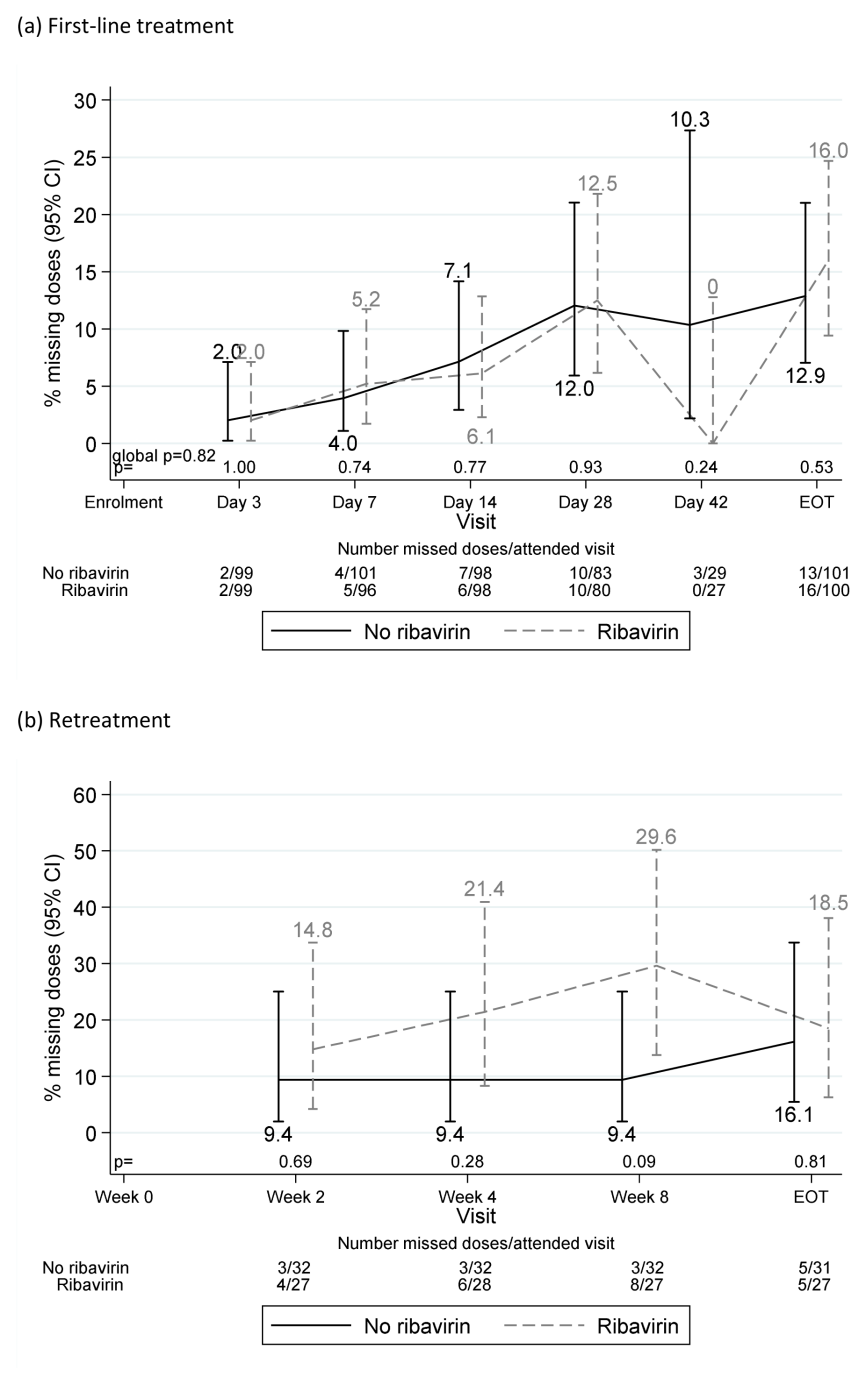
Reported missing doses by ribavirin randomisation. ( **a**) First-line treatment. ( **b**) Retreatment.

Each pre- or post- first-line EOT visit was missed by no more than eight (4%) participants. One participant was lost-to-follow-up at day-28 on first-line and three (all randomised to fixed-duration, two with ribavirin) stopped first-line treatment >1 week early. All other participants completed first-line treatment ( Figure 5). Adverse events caused one participant to stop both first-line DAAs and ribavirin three days early (grade 3 mouth sores), and one ribavirin only two days early (grade 3 anaemia). Self-reported non-adherence to DAAs and/or ribavirin varied from 2–14% across first-line visits ( Figure 6(a)), with 55 (28%) reported missing doses at any first-line visit (40 (20%) at one visit only). Each retreatment visit was missed by at most four (6%) participants, with the exception of week-8 post-retreatment (missed by 10 (16%) participants). Self-reported non-adherence was substantially higher on retreatment ( Figure 6(b)), 12–19% across visits, 24 (39%) at any retreatment visit, 17 (27%) at one retreatment visit only).

### Sustained virological response (SVR)

One participant withdrew consent (retreatment week-4) and a further 13 (6%) participants were lost-to-follow-up (1 on first-line, 9 post first-line EOT, 2 on retreatment, 1 post retreatment EOT; total withdrawn/lost 9 (9%) fixed-duration, 4 (4%) variable duration). However, HCV VL results were available from medical notes for most of those not withdrawing consent, meaning first-line SVR12 and SVR24 could not be ascertained for only 3 (1%) and 6 (3%) participants, respectively (1/2 and 3/3 fixed/variable-duration, respectively), and overall (first-line plus retreatment) SVR12 and SVR24 for only 5 (2%) and 8 (4%) participants (2/3 and 4/4 fixed/variable-duration) respectively.

Overall, all evaluable participants achieved SVR12 (and SVR24) on first-line plus retreatment (primary outcome for fixed vs variable-duration randomisation) (100% (95%CI 96%,100%) in both groups, difference 0% (95%CI (Newcombe) -3.8%,+3.7%), within the pre-specified 4% non-inferiority margin).

**Figure 7.**
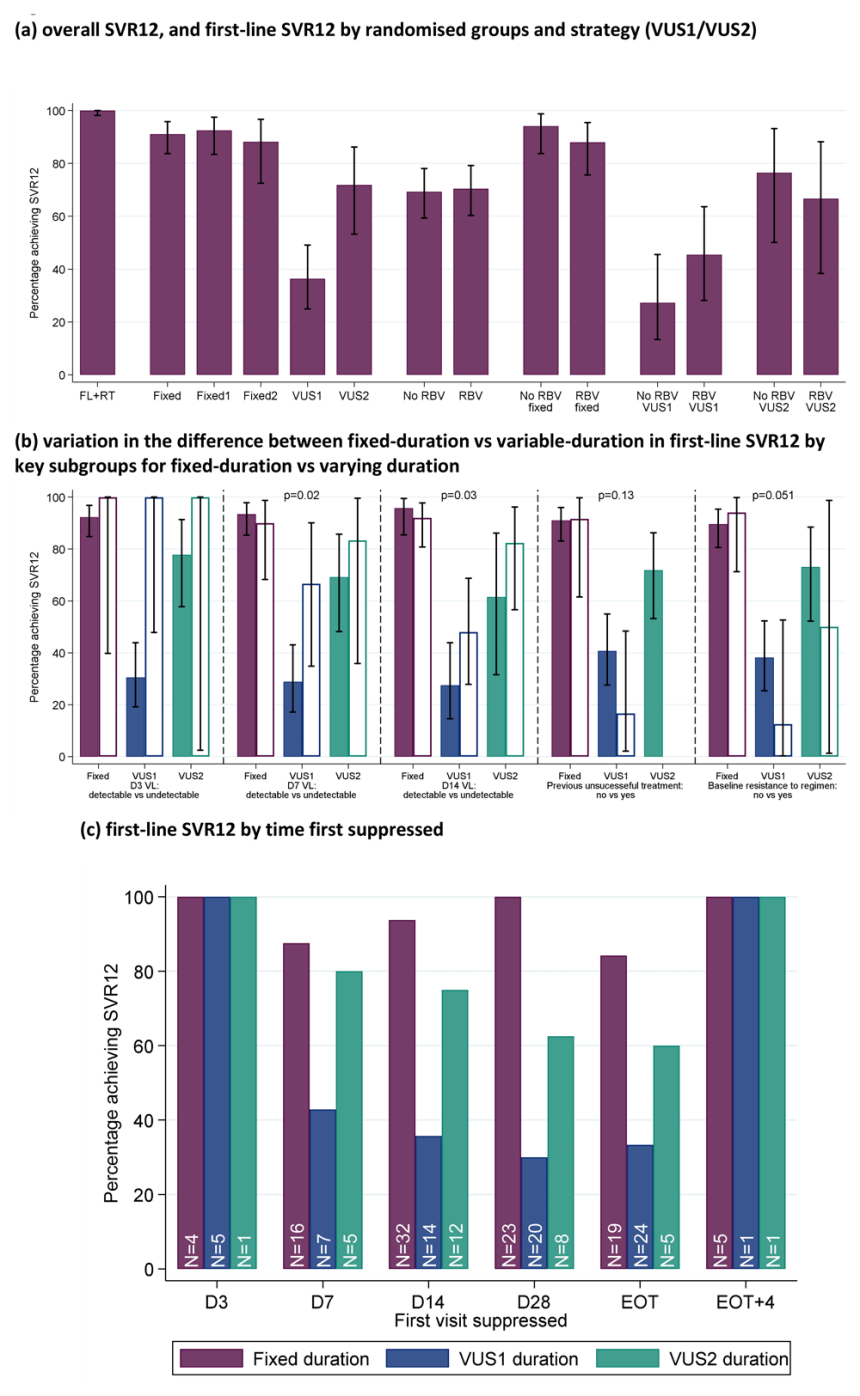
SVR12. ( **a**) overall SVR12, and first-line SVR12 by randomised groups and strategies (VUS1/VUS2). ( **b**) variation in the difference between fixed-duration vs variable-duration in first-line SVR12 by key subgroups. ( **c**) first-line SVR12 by time first suppressed. Note: FL=first-line, RT=retreatment. Fixed = overall SVR12 for 8 week therapy. Fixed1=fixed duration when VUS duration received VUS1, Fixed2=fixed duration when VUS duration received VUS2. In panel ( **b**), solid bars represent the first subgroup (detectable VL at the various days shown, no previous unsuccessful treatment, no baseline resistance to drugs taken first-line) and empty bars the second subgroup (undetectable VL at the various days shown, previous unsuccessful treatment, baseline resistance to drugs taken first-line). p-values are heterogeneity p-values comparing the difference between fixed vs VUS1/VUS2 strategies across the two subgroups. Heterogeneity p-value for D3 VL cannot be estimated due to perfect prediction. Panel (c) does not include 8 patients who never had VL suppression; for patients allocated 28–31 days, their EOT visit is also their 28 day visit and they are included in each group.

First-line SVR12 (secondary outcome) was 91% (95% CI 86%,97%; 92/101) in the fixed-duration group vs 48% (39,57%; 47/98) in the variable-duration group ( Figure 7(a); difference -43% (95% CI -54%,-32%), p<0.0001). However, SVR12 was significantly higher for VUS2 (72% (56%,87%); 23/32) than VUS1 (36% (25%,48%); 24/66) (interaction between duration randomisation and strategy p=0.001). First-line SVR12 was 72% (65%,78%); 70/101) with ribavirin and 68% (61%,76%; 69/98) without (difference -3% (-13%, +6%) p=0.48 adjusting for interaction between duration randomisation and strategy). There was no evidence of interaction between ribavirin and duration randomisations overall (heterogeneity p=0.16 adjusted for the interaction between duration randomisation and strategy) or in the variable-duration group, where SVR12 was 52% (37%,67%; 25/48) with ribavirin vs 44% (30%,59%; 22/50) without ribavirin (difference 8% (95% CI -10%,+27%), p=0.38).

**Figure 8.**
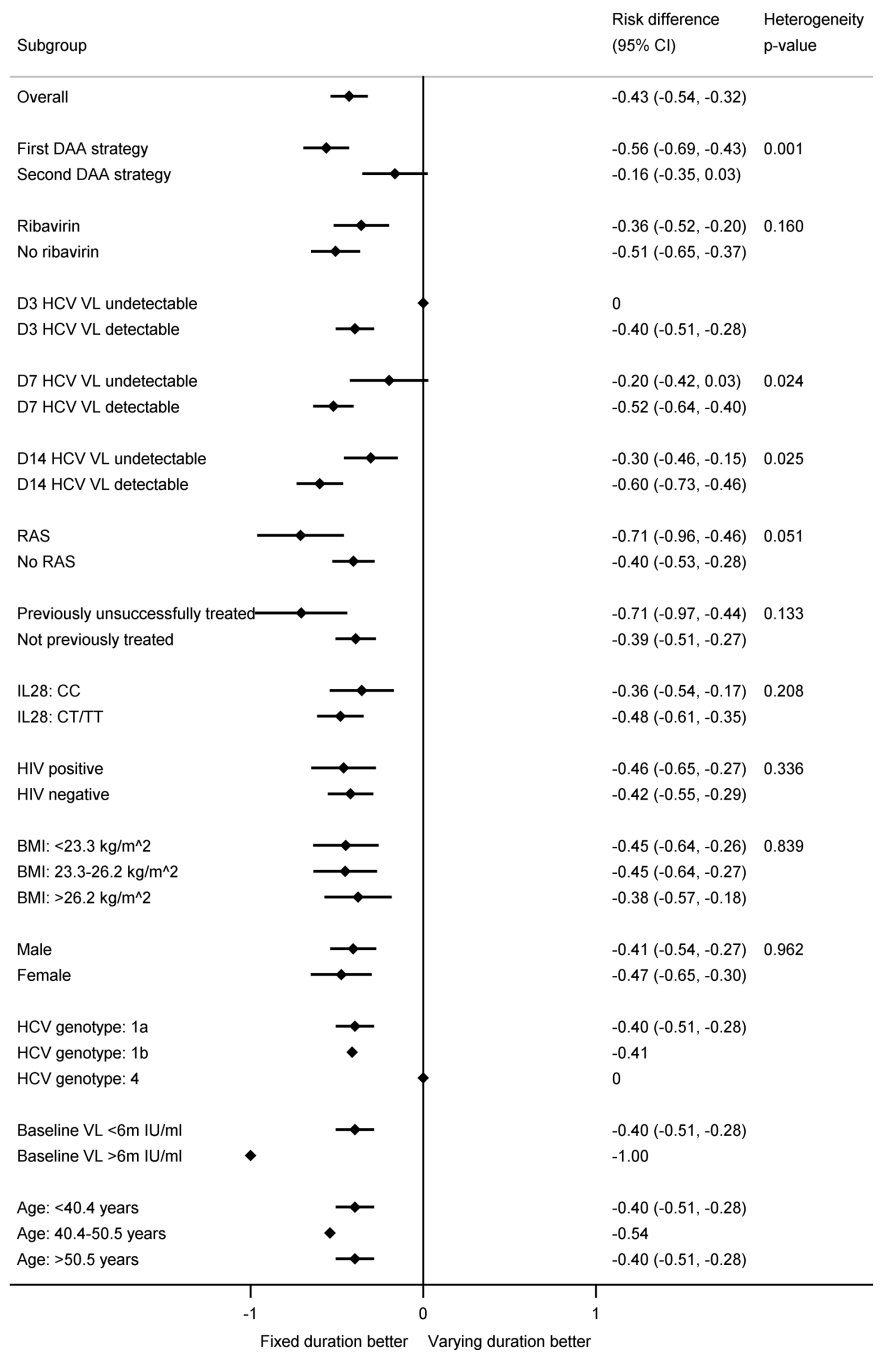
Subgroup analyses for first-line SVR12 by fixed-duration vs variable-duration randomisation. Note: RAS=resistance associated substitution. All heterogeneity tests also adjusted for the interaction between strategy and fixed-duration vs variable-duration randomisation. Some confidence intervals and heterogeneity p-values could not be estimated due to perfect prediction.

**Figure 9.**
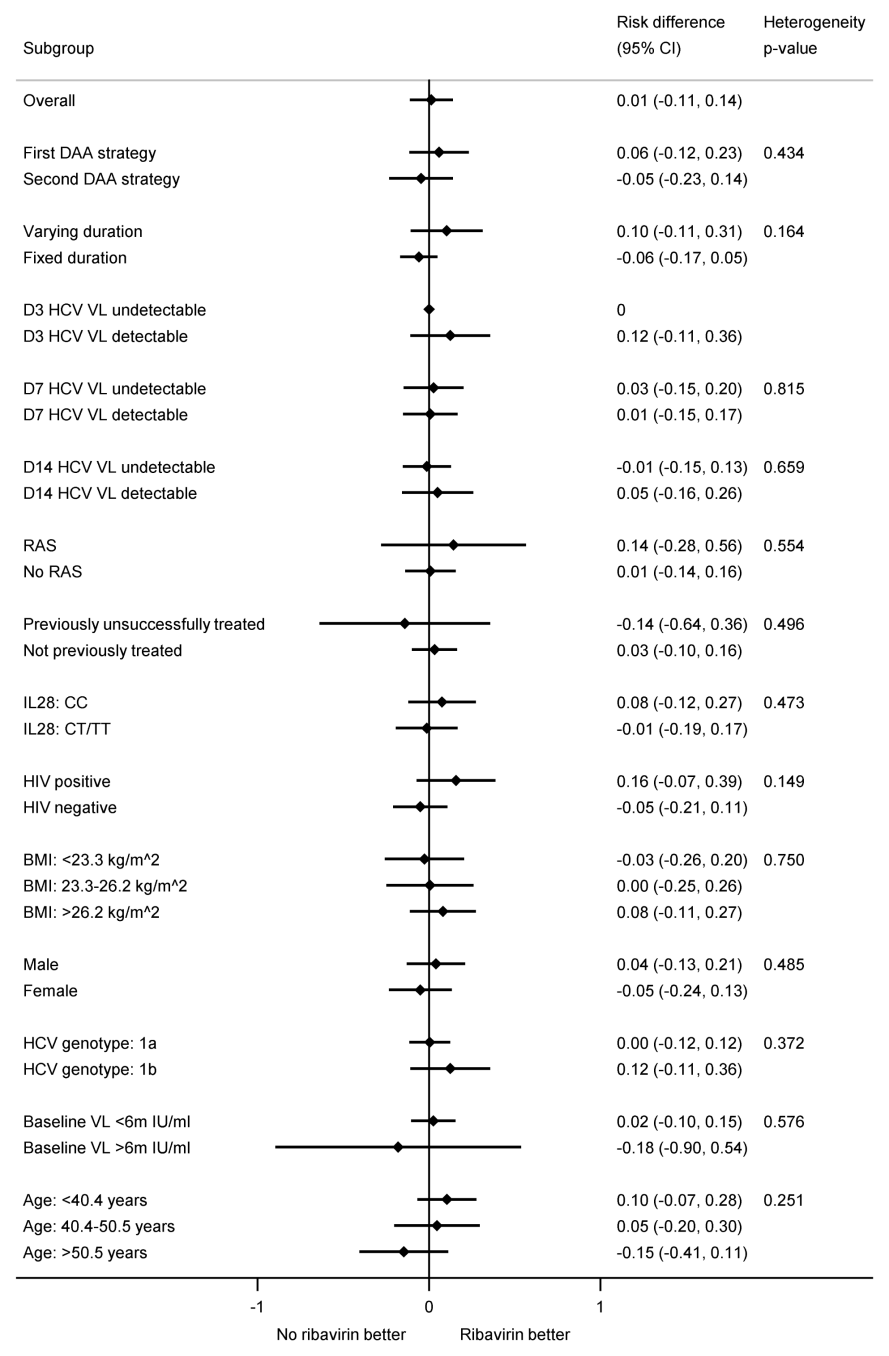
Subgroup analyses for first-line SVR12 by ribavirin randomisation. Note: RAS=resistance associated substitution. All heterogeneity tests also adjusted for the interaction between strategy and fixed-duration vs variable-duration randomisation. Some confidence intervals and heterogeneity p-values could not be estimated due to perfect prediction. All participants with genotype 4 did not receive ribavirin so not included.

The difference in first-line SVR12 between fixed-duration vs variable-duration was significantly smaller in 2 of 16 subgroups pre-specified in the protocol, suppression at day-7 or day-14 (heterogeneity p=0.02, 0.03 respectively) ( Figure 7(b) and Figure 8). Considering the time when individuals first became undetectable , all 10 individuals who became undetectable at day-3 of treatment achieved first-line SVR12 regardless of treatment duration (as did 31/38 (82%) of those first undetectable at day-7) ( Figure 7(c)). In the ribavirin randomisation, no subgroup had a difference that was significantly larger or smaller ( Figure 9).

In total, 70 (70%) receiving VUS1/VUS2 vs 72 (71%) receiving fixed-duration were included in the per-protocol population and 68 (68%) receiving ribavirin vs 74 (73%) not receiving ribavirin (received >90% and <100% of the prescribed duration of first-line treatment and had a difference between screening and enrolment HCV RNA values leading to a difference of ≤2 days in allocated duration of DAAs had they been allocated to the variable-duration group). For the duration randomisation SVR12, after first-line and any retreatment was 100% overall (95% CI 97%, 100%) with a difference of 0% (95% CI (Newcombe) -5%, +5%). SVR12 after first-line only was 47% (95% CI 36%, 59%; 32/69) in the variable-duration group vs 93% (95% CI 87%, 99%; 66/71) in the fixed-duration group. The difference was -46% (95% CI -59%, -33%; p<0.0001). For the ribavirin randomisation, SVR12 after first-line and any retreatment was 100% overall (95% CI 97%, 100%) with a difference of 0% (95% CI (Newcombe) -0.06%, +0.05%). SVR12 after first-line only was 70% (95% CI 61%, 78%; 48/66) in the ribavirin group vs 70% (95% CI 62%, 78%; 50/74) in the no ribavirin group. The difference was -0% (95% CI -11%, +10%; p=0.93).

Results were similar for SVR24. For the duration randomisation, SVR24 after first-line and any retreatment was 100% overall (95% CI 98%, 100%; 194/194) with a difference of 0% (95% CI (Newcombe) -3.8%, +3.8%). SVR24 after first-line only was 47% (95% CI 38%, 56%; 46/97) in the variable-duration group vs 89% (95% CI 83%, 95%; 88/99) in the fixed-duration group. The difference was -42% (95% CI -53%, -31%; p<0.0001). For the ribavirin randomisation, SVR24 after first-line and any retreatment was 100% overall (95% CI 95%, 100%); 194/194) with a difference of 0% (95% CI (Newcombe) -3.8%, 3.8%). SVR24 after first-line only was 69% (95% CI 61%, 76%; 68/97) in the ribavirin group vs 68% (95% CI 60%, 76%; 66/99) in the no ribavirin group. The difference was 1% (95% CI -9%, +11%; p=0.87).

### Timing of failure

**Figure 10.**
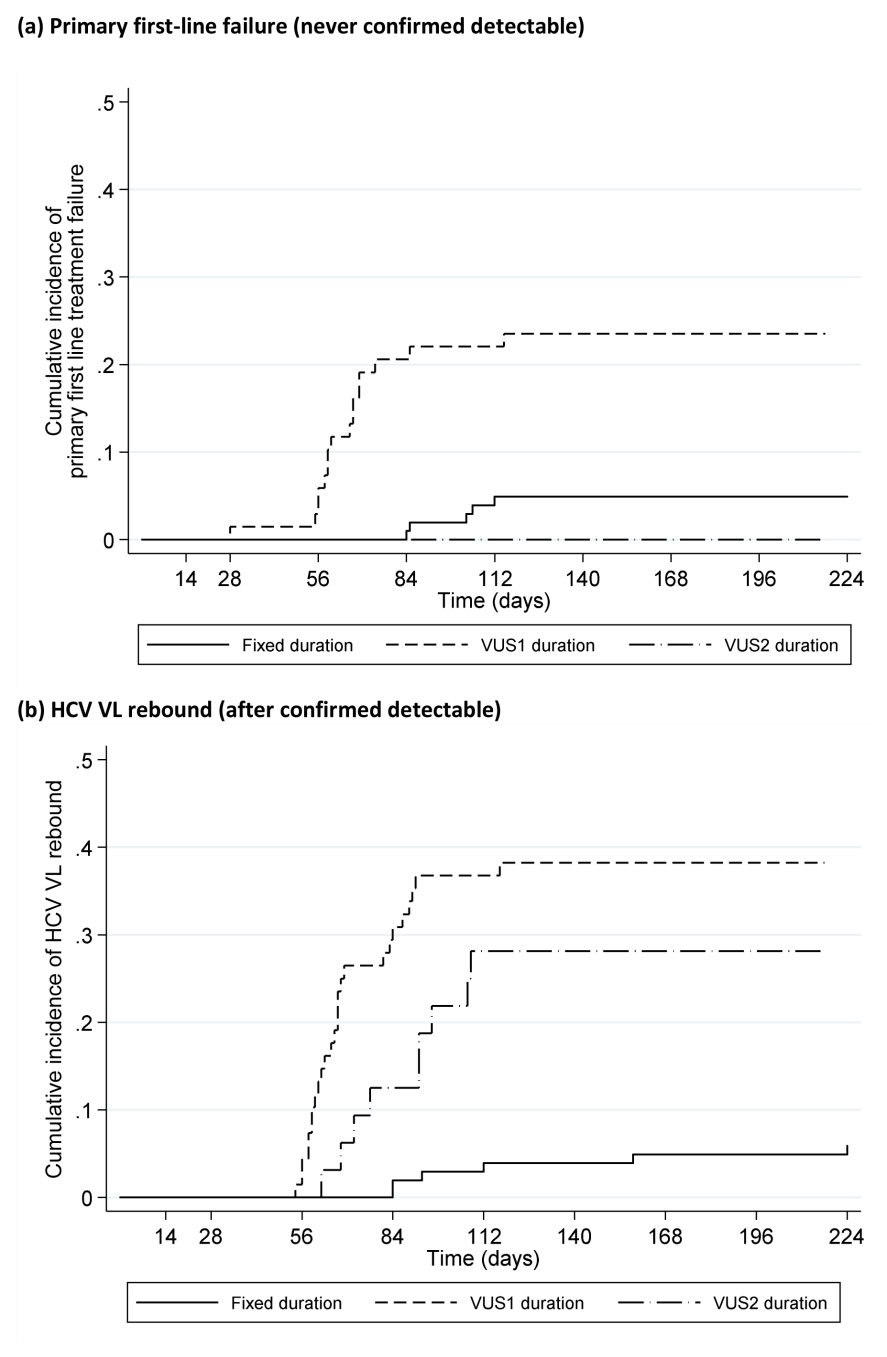
Time to failure on first-line by fixed duration vs variable duration randomisation. ( **a**) Primary first-line failure (never confirmed detectable). ( **b**) HCV VL rebound (after confirmed detectable).

**Figure 11.**
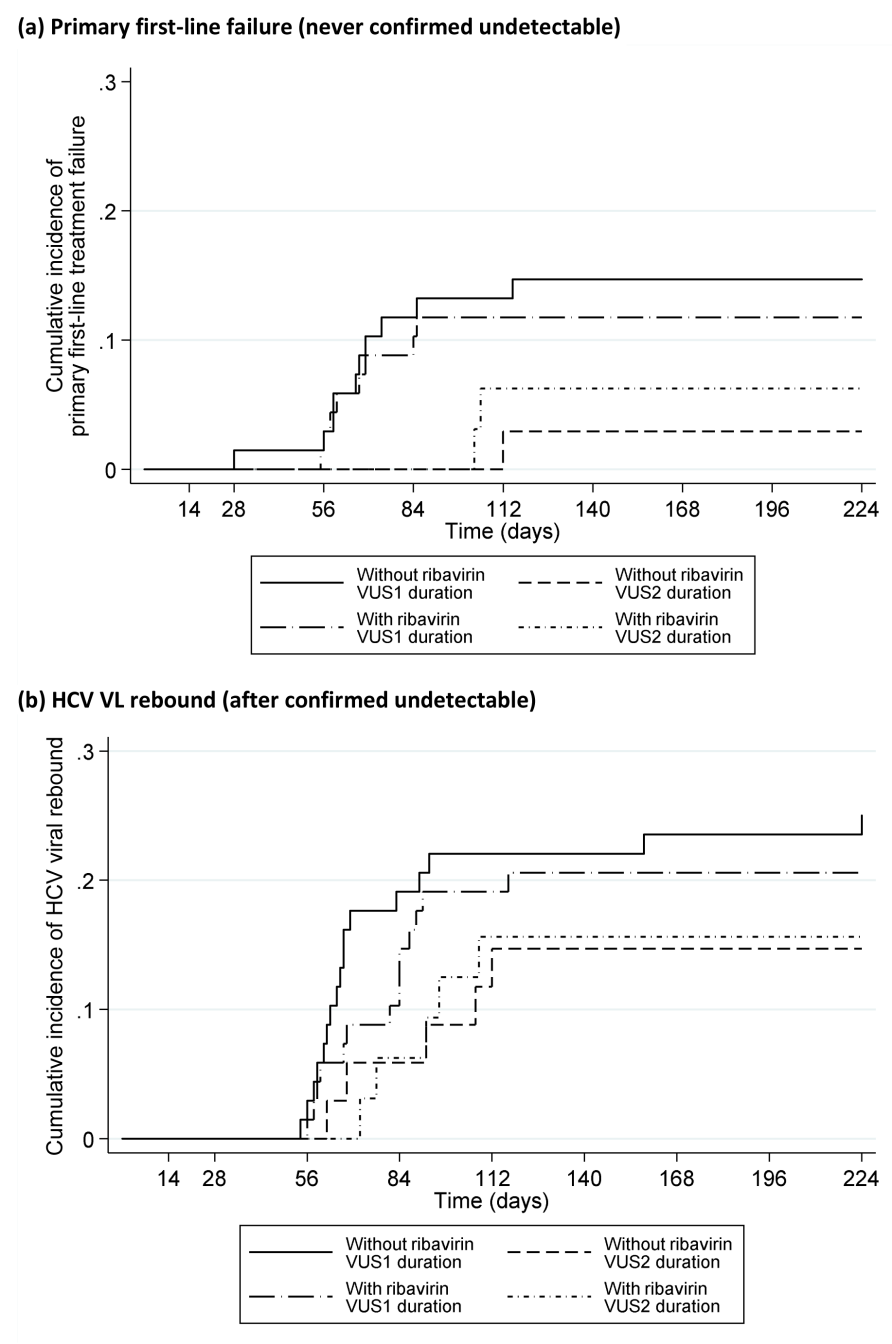
Time to failure on first-line by ribavirin randomisation. ( **a**) Primary first-line failure (never confirmed detectable). ( **b**) HCV VL rebound (after confirmed detectable).

Only 1 (0.5%) (VUS1) participant failed on treatment (at EOT, 28 days DAAs). 21 (10%) participants had primary first-line failure (VL never confirmed undetectable) (5 (5%) fixed-duration vs 16 (16%) variable-duration, p=0.008, Figure 10(a)); in the variable-duration group, primary failure occurred in 16 (24%) VUS1 vs 0 (0%) VUS2 (p=0.002; interaction not estimable across strategies). 41 (20%) participants had VL rebound after confirmed undetectable HCV VL (6 (6%) fixed-duration vs 35 (35%) variable-duration, p<0.0001, Figure 10(b); 26 (38%) VUS1, 9 (28%) VUS2, heterogeneity p=0.60). There was no evidence that ribavirin was associated with primary failure (p=0.83) or rebound (p=0.59) ( Figure 11). Failure tended to occur earlier with VUS1 vs VUS2 (p=0.08), and with variable-duration vs fixed-duration (p=0.07; VUS1 vs fixed-duration p=0.03). However, there was no evidence of differences in failure VLs (median 158073 (VUS1), 89125 (VUS2), 346737 (fixed-duration), p(VUS2 vs VUS1)=0.41, p(VUS2 vs fixed-duration)=0.21). All participants who met failure criteria on first-line started retreatment, a median (IQR) 2.9 (2.0,4.4) weeks after failure was confirmed.

**Figure 12.**
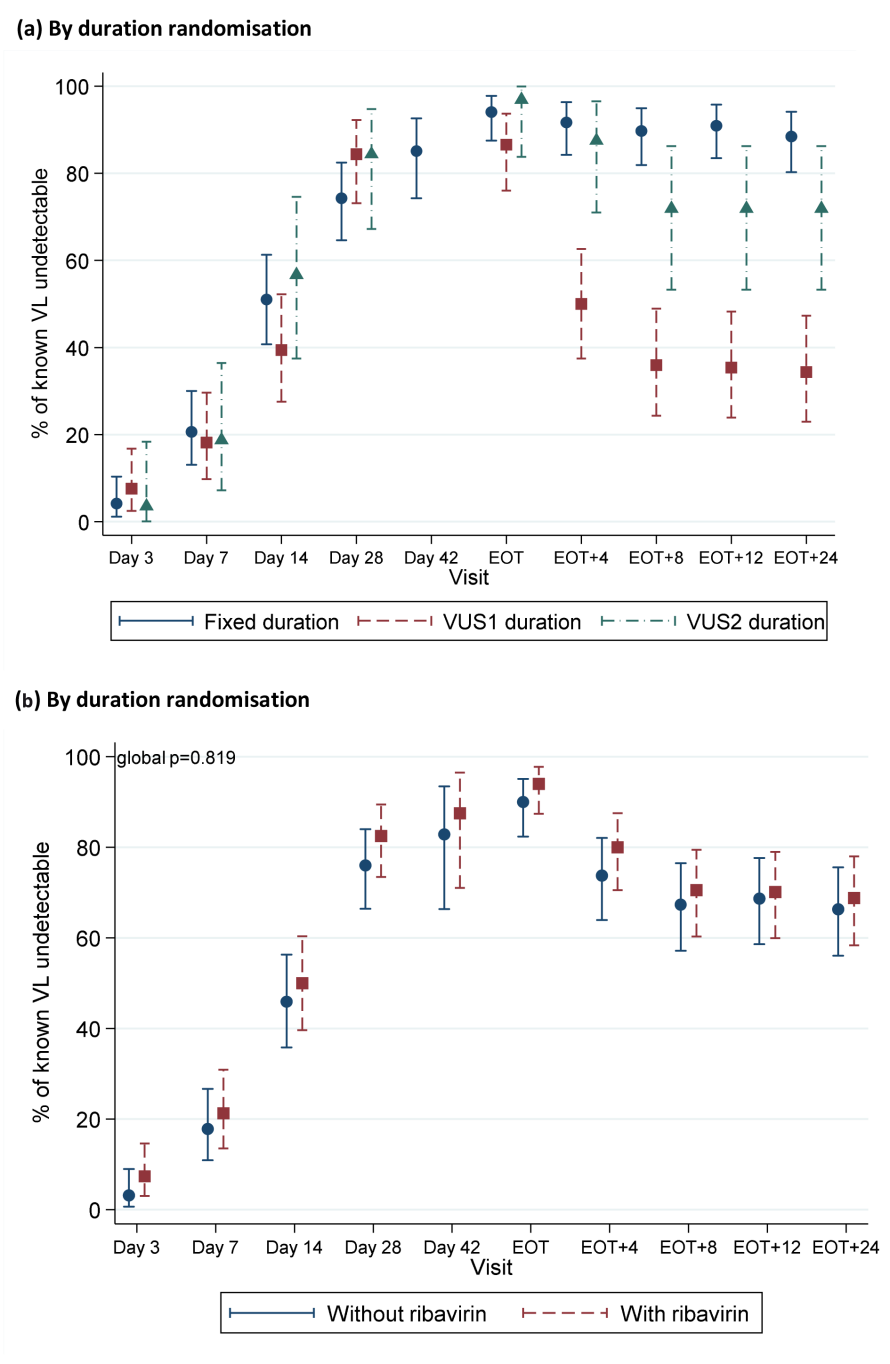
HCV VL suppression first-line. ( **a**) By duration randomisation. Note: EOT=end of treatment. Carrying forward last detectable value for participants meeting failure criteria. No evidence of difference between groups through day 28 when all participants were receiving DAAs (global p=0.13 comparing fixed-duration vs variable-duration combined; p=0.10 and 0.82 comparing VUS1 and VUS2 vs fixed, respectively). Post-EOT global p<0.0001 comparing fixed-duration vs variable-duration combined; p<0.0001 and 0.53 comparing VUS1 and VUS2 vs fixed, respectively. ( **b**) By ribavirin randomisation. Note: EOT=end of treatment. Carrying forward last detectable value for participants meeting failure criteria. No evidence of difference between groups through day 28 when all participants were receiving DAAs (global p=0.62 comparing ribavirin vs no ribavirin (combining fixed-duration vs variable-duration combined); interaction p=0.28). Post-EOT global p=0.48 comparing ribavirin vs no ribavirin (combining fixed-duration vs variable-duration combined; interaction p=0.22).

Corresponding to the timing of failures, the percentages with undetectable VL decreased more rapidly post-EOT in VUS1 vs VUS2 (p<0.0001) or VUS1 vs fixed-duration (p<0.0001) ( Figure 12(a)). There was no evidence of differences in the proportions with detectable VL at EOT in VUS1 compared to VUS2 (p=0.33) or fixed-duration (p=1.00). There was no evidence that ribavirin was associated with higher percentages detectable post-EOT overall (p=0.48) or, within the variable-group, between VUS1 vs VUS2 (interaction p=0.17, Figure 12(b)).

### Emergence of resistance

**Figure 13.**
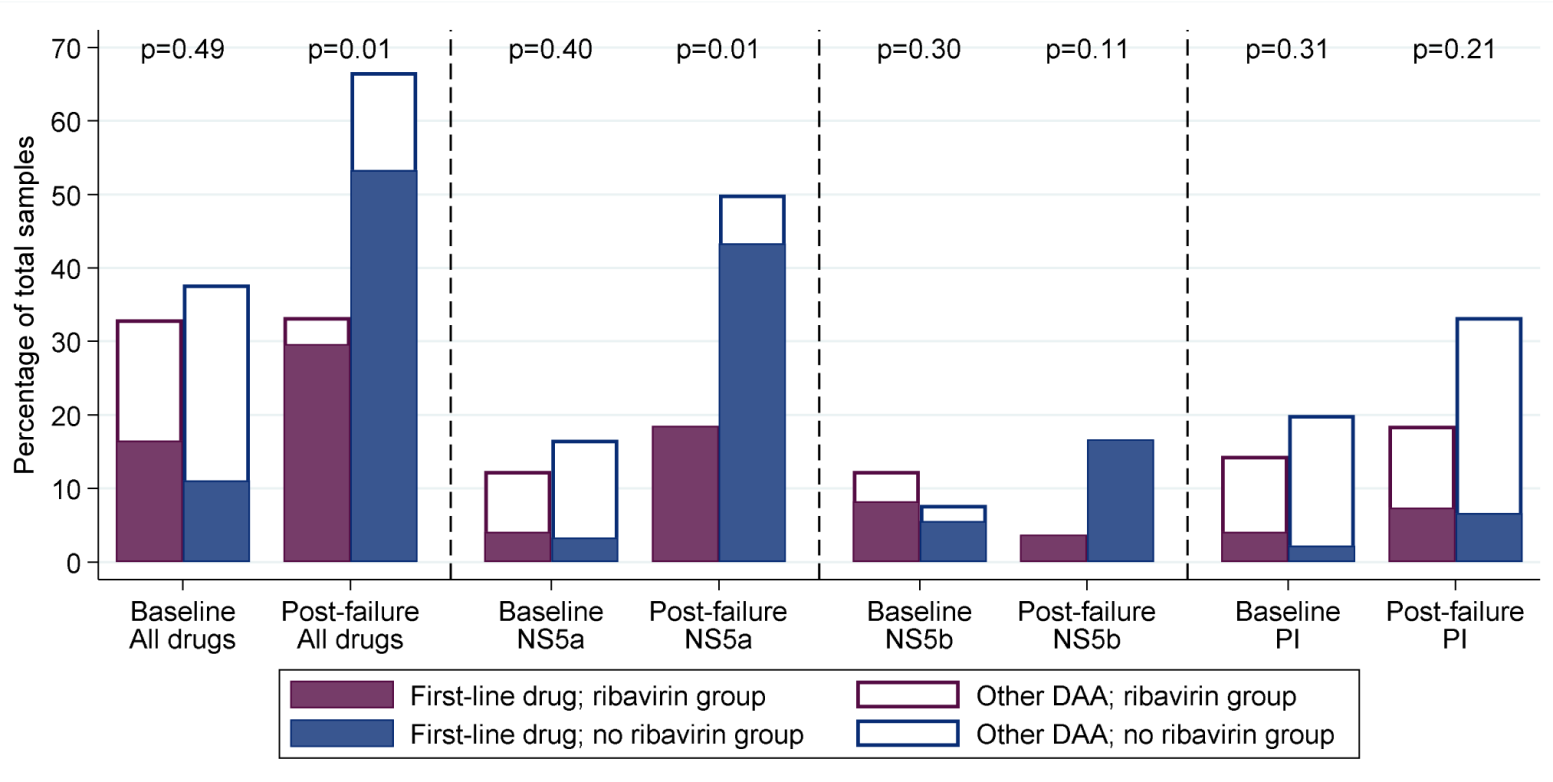
Resistance at baseline (all tested participants) and at first-line failure (failures only). Note: DAA: direct acting antivirals. p-values are comparing any resistance, to first-line drug or any other DAA, between ribavirin groups. Coloured bars represent resistance to drugs received first-line, white bars, all DAAs including those not used in the trial.

In total, 14 participants who failed on first-line treatment developed a new resistance mutation (not present at baseline) to at least one of their prescribed drugs ( Table 4, Table 5). Within paired samples available at baseline and failure, there was no evidence of a difference in emergent resistance between fixed-duration and variable-duration (3/10 (30%) vs 11/46 (24%, respectively, p=0.77). However, ribavirin was associated with lower emergence of resistance to first-line drugs (3/27 (11%) with vs 11/29 (38%) without ribavirin, p=0.01; Figure 13) with significantly lower rates of resistance to any DAA and to NS5a inhibitors (both p=0.01).

**Table 4.** Summary of RAS to any DAA in genotype 1a by time point.

	Baseline	Post-failure
Resistance to NS5a inhibitors	N=13	N=18
24: K24R	1 (8%)	2 (11%)
28: M28T	-	5 (28%)
M28V	6 (46%)	5 (28%)
30: Q30H	1 (8%)	-
Q30L	-	1 (6%)
Q30R	-	6 (33%)
31: L31M	-	2 (11%)
58: H58D	-	2 (11%)
93: Y93F	1 (8%)	-
Y93H	-	1 (6%)
Y93N	1 (8%)	1 (6%)
Resistance to NS5b inhibitors	N=7	N=3
448: Y448H	2 (29%)	-
556: S556G	5 (71%)	3 (100%)
Resistance to protease inhibitors	N=30	N=14
36: V36M	1 (3%)	1 (7%)
55: V55A	3 (10%)	2 (14%)
V55I	3 (10%)	1 (7%)
80: Q80K	21 (70%)	9 (64%)
Q80L	1 (3%)	-
155: R155K	-	1 (7%)
168: D168A	-	1 (7%)

**Table 5.** Summary of RAS to any DAA genotype in 1b by time point.

	Baseline	Post failure
Resistance to NS5a inhibitors	N=13	N=2
30: R30Q	1 (8%)	-
31: L31I	1 (8%)	-
L31M	1 (8%)	-
37: L37I	1 (8%)	-
54: Q54H	11 (84%)	2 (100%)
58: P58S	1 (8%)	-
Resistance to NS5b inhibitors	N=10	N=3
159: L159F	5 (50%)	1 (33%)
316: C316H	2 (20%)	1 (33%)
C316N	4 (40%)	1 (33%)
556: S556G	6 (60%)	3 (100%)
Resistance to protease inhibitors	N=2	N=1
122: S122N	1 (50%)	1 (100%)
168: D168E	1 (50%)	-

### Adverse events

Overall, five (5%) variable-duration vs five (5%) fixed-duration participants experienced serious adverse events (SAEs) (hazard ratio (HR)=0.77 (95% CI 0.21,2.80) p=0.69) and nine (9%) vs five (5%) respectively experienced Grade 3/4 adverse events (AEs) (HR=1.74 (0.58,5.24) p=0.33) ( Table 6, Table 7). Similarly, five (5%) ribavirin vs five (5%) no ribavirin participants experienced SAEs (HR=1.05 (95% CI 0.30,3.63) p=0.59) and nine (9%) vs five (5%) respectively experienced Grade 3/4 AEs (HR=1.92 (0.64,5.72) p=0.59) ( Table 7, Table 8). Treatment-related AEs, AEs causing changes to treatment and Grade 3/4 anaemia were all uncommon ( Table 6); all Grade 3/4 anaemias occurred in participants randomised to adjunctive ribavirin (p=0.12) as did all first-line drug changes for AEs (p=0.06) ( Table 8). 

**Table 6.** Summary of adverse events by fixed duration vs variable duration randomisation.

	Variable-duration	Fixed-duration	Total	p-value *
Number randomised	N=100	N=102	N=202	
Median weeks follow-up (IQR)	49.0 (29.0, 54.4)	32.0 (32.0, 33.0)	32.1 (31.2, 50.4)	
**SAEs**	**5 (5%) [5]**	**5 (5%) [5]**	**10 (5%) [10]**	**p=1.00**
Life-threatening	1 (1%) [1]	1 (1%) [1]	2 (1%) [2]	
Required or prolonged hospitalisation	5 (5%) [5]	4 (4%) [4]	9 (4%) [9]	
Other important medical condition	0	1 (1%) [1]	1 (<1%) [1]	
Relationship to trial drug (% of SAEs)				
Unlikely	2 (40%)	2 (40%)	4 (40%)	
Not related	3 (60%)	3 (60%)	6 (60%)	
**Severe AEs**	**9 (9%) [16]**	**5 (5%) [5]**	**14 (7%) [21]**	**p=0.28**
Relationship to trial drug (% of severe AEs)				
Definitely	8 (50%)	0	8 (38%)	
Probably	2 (13%)	2 (40%)	4 (19%)	
Possibly	3 (19%)	0	3 (14%)	
Unlikely	0	1 (20%)	1 (5%)	
Not related	3 (19%)	2 (40%)	5 (24%)	
**AEs probably/definitely related to first-line drugs**	**3 (3%) [3]**	**1 (1%) [1]**	**4 (2%) [4]**	**p=0.37**
**AEs probably/definitely related to retreatment drugs**	**3 (3%) [7]**	**1 (1%) [1]**	**4 (2%) [8]**	**p=0.37**
**First-line drug changes due to AEs**	**3 (3%) [3]**	**1 (1%) [1]**	**4 (2%) [4]**	**p=0.37**
**Retreatment drug changes due to AEs**	**6 (6%) [11]**	**1 (1%) [1]**	**7 (3%) [12]**	**p=0.06**
**Grade 3/4 anaemia**	**3(3%) [3]**	**0**	**3 (1%) [3]**	**p=0.12**

*p-values calculated using chi-square tests or Fishers exact test when numbers are small.

Note: no. of patients (% of patients) [no. of events]. Tables include data for both first-line and retreatment phases. For SAEs, HR=0.77 (0.21, 2.80) p=0.69. For severe (grade 3/4 AEs), HR=1.74 (0.58, 5.24) p=0.33.

**Table 7.** Details of adverse events.

Event	Variable, ribavirin	Variable, no ribavirin	Fixed, ribavirin	Fixed, no ribavirin	Total Events
**SAEs**					
Accidental drug overdose *	1	0	0	0	1
Acute appendicitis	0	0	0	1	1
Adenocarcinoma in lower third of oesophagus *	0	0	0	1	1
Burn to foot - degree unknown	1	0	0	0	1
Liver abscess	1	0	0	0	1
Lower respiratory tract infection - pneumonia	1	0	0	0	1
Musculoskeletal pain in chest radiating to left arm	0	0	0	1	1
Pericarditis	1	0	0	0	1
R epididymo-orchitis	0	0	0	1	1
Urinary sepsis	0	0	0	1	1
**Severe AEs**					
Abscess leg	01	0	0	0	1
Alcohol intoxication acute	0	0	0	1	1
Anaemia	2	0	0	0	2
Cellulitis of leg	0	1	1	0	2
Concentration loss	1	0	0	0	1
Haemoglobin low	1	0	0	0	1
Hyperbilirubinemia	0	1	0	0	1
Inguinal hernia	1	0	0	0	1
Insomnia	3	0	0	0	3
Jaundice	0	0	0	1	1
Lethargic	1	0	0	0	1
Low mood	1	0	0	0	1
Pyelonephritis	0	0	1	0	1
Sores mouth	1	0	0	0	1
Suicidal ideation	1	0	0	0	1
Syncope	0	0	0	1	1
Tinnitus	1	0	0	0	1
**AEs probably/definitely related to trial drugs**					
Anaemia	2	0	0	0	2
Concentration loss	1	0	0	0	1
Haemoglobin low	1	0	0	0	1
Hyperbilirubinemia	0	1	0	0	1
Insomnia	3	0	0	0	3
Jaundice	0	0	0	1	1
Lethargic	1	0	0	0	1
Low mood	1	0	0	0	1
Syncope	0	0	0	1	1
Tinnitus	1	0	0	0	1
**Drug changes due to AEs**					
Anaemia	3	3	0	0	6
Concentration loss	1	0	0	0	1
Haemoglobin low	1	0	1	0	2
Hair loss	0	0	1	0	1
Hyperbilirubinemia	0	1	0	0	1
Insomnia	1	0	0	0	1
Lethargic	1	0	0	0	1
Low mood	1	0	0	0	1
Mouth ulcer	1	0	0	0	1
Sores mouth	1	0	0	0	1

*Life-threatening events

**Table 8.** Summary of adverse events by ribavirin randomisation.

	With ribavirin	Without ribavirin	Total	p-value *
Number randomised	N=100	N=102	N=202	
Median weeks follow-up (IQR)	32.1 (30.4, 50.4)	32.1 (31.9, 49.0)	32.1 (31.2, 50.4)	
**SAEs**	**5 (5%) [5]**	**5 (5%) [5]**	**10 (5%) [10]**	**p=1.00**
SAE criteria				
Life-threatening	1 (1%) [1]	1 (1%) [1]	2 (1%) [2]	
Required or prolonged hospitalisation	5 (5%) [5]	4 (4%) [4]	9 (4%) [9]	
Other important medical condition	0	1 (1%) [1]	1 (<1%) [1]	
Relationship to ribavirin (% of SAEs)				
Unlikely	2 (40%)	2 (40%)	4 (40%)	
Not related	3 (60%)	3 (60%)	6 (60%)	
**Severe AEs**	**9 (9%) [15]**	**5 (5%) [6]**	**14 (7%) [21]**	**p=0.28**
Relationship to trial drug (% of severe AEs)				
Definitely	8 (53%)	0	8 (38%)	
Probably	1 (7%)	3 (50%)	4 (19%)	
Possibly	3 (20%)	0	3 (14%)	
Unlikely	1 (7%)	0	1 (5%)	
Not related	2 (13%)	3 (50%)	5 (24%)	
**AEs probably/definitely related to first line drugs**	**3 (3%) [3]**	**1 (1%) [1]**	**4 (2%) [4]**	**p=0.37**
**AEs probably/definitely related to retreatment drugs**	**2 (2%) [6]**	**2 (2%) [2]**	**4 (2%) [8]**	**p=1.00**
**First line drug changes due to AEs**	**4 (4%) [4]**	**0**	**4 (2%) [4]**	**p=0.06**
**Retreatment drug changes due to AEs**	**4 (4%) [8]**	**3 (3%) [4]**	**7 (3%) [12]**	**p=0.72**
**Grade 3/4 anaemia**	**3(3%) [3]**	**0**	**3 (1%) [3]**	**p=0.12**

*p-values calculated using chi-square tests or Fishers exact test when numbers are small.

Note: no. of patients (% of patients) [no. of events]. Tables include data for both first-line and retreatment phases. For SAEs, HR=1.05 (95% CI 0.30, 3.63) p=0.94. For severe (grade 3/4 AEs), HR=1.92 (0.64, 5.72) p=0.59.

## Discussion

This large strategic post licensing trial demonstrated overall non-inferiority of strategies using first-line ultrashort treatment durations, with both variable duration and fixed duration groups achieving 100% SVR12 rate after retreatment. The initial shortening strategy (VUS1) was able to cure only 36% of participants first-line but, strikingly, a relatively small increase in ultrashort treatment duration (from a mean of 32 days to 39 days) resulted in a doubling of SVR12 rates (from 36% to 72%). The 8-week fixed-duration strategy, with an SVR12 of 91%, did not have higher efficacy than previous phase II trials of shorter treatment courses ^19^, despite limiting the maximal baseline viral load in those enrolled, which might have been expected to reduce the risk of failure. The trial’s findings suggest a high proportion of patients can be cured with, on average, approximately 60% of the licensed duration of first-line therapy with the agents used in the trial (VUS2 strategy). However, first-line cure rates were not sufficient to be routinely recommended for stable patients able to adhere to 8–12 weeks’ therapy.

Previous work has found adherence to DAA therapy declines as treatment progresses, with patients citing “feeling as if the treatment is working” as a reason for decreasing adherence ^5^. Despite trial participants generally being considered to have better adherence, we found similarly decreasing adherence with time on first-line, with 28% reporting missed first-line doses, and poorer adherence to retreatment (despite its 100% SVR12) ( Figure 5). That we observed 72% SVR12 with VUS2 (mean duration 39 days) despite 28% reporting missing doses, suggests that intermittent non-adherence may be less important than overall adherence during weeks 4 to 8 of first-line treatment, and emphasise the importance of supporting adherence after week 4 of therapy to ensure good cure rates in hard-to-reach populations ^10, 20^.

The risk of virological failure, in a patient unlikely to complete the recommended treatment course, is a clinical concern. Emergent resistance could compromise retreatment, particularly where retreatment does not include a protease inhibitor (as in this trial, in contrast to licensed retreatment options). This is also an ethical consideration in short-course therapy trials. We found the first-line treatment strategy did not compromise participants’ ability to ultimately achieve SVR12. Whether this would be the case with pan-genotypic first-line treatment regimens remains to be tested, although it seems plausible. The 100% SV12 rates for retreatment is reassuring from an ethical perspective and suggests that, in certain circumstances, the combination may represent a viable retreatment option for patients failing therapy where access to licensed retreatment options (such as sofosbuvir/velpatasvir/voxilaprevir) remains limited.

Although ribavirin side-effects, particularly anaemia and fatigue, increasingly limit its use ^2, 12^ it still has a role for some patients ^13^ with limited evidence that it can increase efficacy ^21^ and *in vitro* evidence that it may reduce the emergence of resistance with short course therapy ^22^. Here, additional ribavirin was well tolerated, with only 2–4% participants experiencing adverse events. Across randomised groups, there was no evidence of improvement in SVR12. However, the emergence of resistance was significantly lower in those failing therapy (12% with ribavirin v 38% without), the first time this has been demonstrated in a randomised trial. This suggests that adjunctive ribavirin may have a role for some patients considered at high risk of not completing therapy in order to reduce the risk of compromising retreatment and further work is required to understand better the mechanism of action in this setting.

Response-guided therapy, shortening treatment based on initial virological response, was commonplace for interferon-based therapy ^23^ but this approach is not currently recommended for DAA therapy ^24, 25^. Our findings suggest, for the first time, that very early responses to treatment (undetectable at day-3, or even day-7) may be helpful in predicting success of shortened treatment courses. Whilst not widely applicable, in specific supervised clinical settings (including in-patients, prisoners or directly observed daily therapy in the community) such an approach may help guide management and deserves further confirmation in prospective studies ^25– 27^.

This trial was designed to test treatment strategies, rather than specific regimens. Almost all recruitment happened when ombitasvir, paritaprevir, dasabuvir and ritonavir (Viekirax, Abbvie) was the preferred first-line treatment in the UK National Health Service (NHS). This combination remains a recommended NHS option, part of the WHO Essential Medicines List and is used in a number of countries. However, its use has been superceded by pangenotypic options in many settings and the extent to which these findings can be generalised to other combinations with broader genotype coverage is unknown. Give the similar declines in HCV VL between this and other DAA combinations ( Figure 12), it seems plausible that the relationship between treatment duration and SVR12 is similar for other DAAs combinations approved for 12 weeks for patients with mild disease.

The trial required a population able to adhere to a schedule with significantly more visits than standard of care. Non-attendance was low and self-reported adherence reasonably high ( Figure 5), despite one-third participants actively using recreational drugs. Following expanded access to DAA therapy, trial recruitment completed short of its original target when there were very few patients in need of treatment able to adhere to the follow-up schedule. However, higher than anticipated success of retreatment (predicted to be 85%, actually 100%) and lower than expected loss to follow-up meant that the trial was able to demonstrate non-inferiority according to its pre-specified margin, providing confidence that either strategy would result in an overall SVR12 rate of at least 96% (higher than originally specified).

The treatment of individuals who are unlikely to complete recommended treatment courses is crucial for elimination strategies. Our findings suggest that ultrashort-courses of treatment can cure a significant proportion of patients with mild liver disease, without compromising retreatment in those not cured. Additional ribavirin in those unlikely to complete a course of treatment may be helpful to prevent the emergence of resistant virus.

## Data availability

### Underlying data

Figshare: STOP-HCV-1 trial data. https://doi.org/10.6084/m9.figshare.14141411.v1 ^18^

This project contains the following underlying data:

- figsharedata.csv (Raw dataset containing baseline demographics, outcomes and VL results)

### Extended data

Figshare: STOP-HCV-1 supplementary material. https://doi.org/10.6084/m9.figshare.14229212.v1 ^17^

This project contains the following extended data:

- STOP-HCV-1 supplementary material.docx (Supplementary methods and results)

### Reporting guidelines

Figshare: CONSORT checklist for ‘Strategic treatment optimization for HCV (STOPHCV1): a randomised controlled trial of ultrashort duration therapy for chronic hepatitis C’. https://doi.org/10.6084/m9.figshare.14216063.v1 ^14^

Data are available under the terms of the Creative Commons Zero "No rights reserved" data waiver (CC0 1.0 Public domain dedication).
