# Supplementary material for: Strategic treatment optimization for HCV (STOPHCV1): a randomised controlled trial of ultrashort duration therapy for chronic hepatitis C
Source: Wellcome Open Res. 2021 Jul 29;6:93. Originally published 2021 Apr 29. [Version 2] doi: 10.12688/wellcomeopenres.16594.2 (PMC8361811; doi:10.12688/wellcomeopenres.16594.2)
Supplement: Peer review history

# Peer review: Reviewer response for version 2

**DOI:** 10.21956/wellcomeopenres.18848.r45219

**Published:** 2021-08-12

**Authors:** George Lau

**Affiliations:** 1 The Humanity & Health Medical Centre, Hong Kong, Hong Kong

I am with satisfied the response.

Is the work clearly and accurately presented and does it cite the current literature?

Partly

If applicable, is the statistical analysis and its interpretation appropriate?

Yes

Are all the source data underlying the results available to ensure full reproducibility?

Yes

Is the study design appropriate and is the work technically sound?

Yes

Are the conclusions drawn adequately supported by the results?

Partly

Are sufficient details of methods and analysis provided to allow replication by others?

Yes

Reviewer Expertise:

Hepatologist.

I confirm that I have read this submission and believe that I have an appropriate level of expertise to confirm that it is of an acceptable scientific standard.

# Peer review: Reviewer response for version 2

**DOI:** 10.21956/wellcomeopenres.18848.r45220

**Published:** 2021-08-03

**Authors:** Kittiyod Poovorawan

**Affiliations:** 1 Mahidol-Oxford Tropical Medicine Research Unit, Mahidol University, Bangkok, Thailand; 2 Department of Clinical Tropical Medicine, Faculty of Tropical Medicine, Mahidol University, Bangkok, Thailand

I have no further comments.

Is the work clearly and accurately presented and does it cite the current literature?

Yes

If applicable, is the statistical analysis and its interpretation appropriate?

Yes

Are all the source data underlying the results available to ensure full reproducibility?

Yes

Is the study design appropriate and is the work technically sound?

Yes

Are the conclusions drawn adequately supported by the results?

Yes

Are sufficient details of methods and analysis provided to allow replication by others?

Yes

Reviewer Expertise:

Gastroenterologist, Viral hepatitis.

I confirm that I have read this submission and believe that I have an appropriate level of expertise to confirm that it is of an acceptable scientific standard.

# Peer review: Reviewer response for version 1

**DOI:** 10.21956/wellcomeopenres.18288.r43636

**Published:** 2021-05-14

**Authors:** George Lau

**Affiliations:** 1 The Humanity & Health Medical Centre, Hong Kong, Hong Kong

Cooke *et al.* performed an excellent non-industrial sponsored study to examine the use of ultra-short variable ultrashort- course treatment strategy (VUS) or fixed 56 days of pan-oral DAAs therapy for CHC GT1/4 patients.

 Major comments:

1. With the rapid change in treatment paradigm from interferon-based to pan-oral DAAs therapy, key issues related CHC treatment have never been fully addressed - no RCT comparison with previous registered SOC (PR therapy) in terms of viral kinetic response, short and long-term virological and clinical outcome (liver-related morbidity and mortality). Indeed, “one-size fit all” versus personalized approach was adopted due to lack of data on the latter approach. In addition, the cost-effectiveness of response-guided shortening of therapy versus longer fixed duration of therapy with re-treatment if failed, have never been properly addressed. Can the author have a cost estimate of VUSs and standard therapy? Long-term follow-up clinical outcome data will be interesting and could be included in the discussion.
2. In the discussion, the rationale of different treatment duration based on baseline viral load needs to be explained, as compared to viral response to DAAs during therapy. This is because baseline VL is not the only factor that affect treatment response. The viral kinetic response might be a more important factor to predict SVR with VUS.

Is the work clearly and accurately presented and does it cite the current literature?

Partly

If applicable, is the statistical analysis and its interpretation appropriate?

Yes

Are all the source data underlying the results available to ensure full reproducibility?

Yes

Is the study design appropriate and is the work technically sound?

Yes

Are the conclusions drawn adequately supported by the results?

Partly

Are sufficient details of methods and analysis provided to allow replication by others?

Yes

Reviewer Expertise:

Hepatologist.

I confirm that I have read this submission and believe that I have an appropriate level of expertise to confirm that it is of an acceptable scientific standard, however I have significant reservations, as outlined above.

# Response

**Published:** 2021-07-11

**Authors:** Graham Cooke

**Affiliations:** Imperial College London, UK

**Reviewer 2**

**Comment: **With the rapid change in treatment paradigm from interferon-based to pan-oral DAAs therapy, key issues related CHC treatment have never been fully addressed - no RCT comparison with previous registered SOC (PR therapy) in terms of viral kinetic response, short and long-term virological and clinical outcome (liver-related morbidity and mortality). Indeed, “one-size fit all” versus personalized approach was adopted due to lack of data on the latter approach. In addition, the cost-effectiveness of response-guided shortening of therapy versus longer fixed duration of therapy with re-treatment if failed, have never been properly addressed. Can the author have a cost estimate of VUSs and standard therapy? Long-term follow-up clinical outcome data will be interesting and could be included in the discussion

**Response:** As discussed, further, more detailed work is planned on the cost-effectiveness of different strategies. Increasingly, health systems are entering into pricing arrangement with originator companies that mean a linear relationship between treatment duration and price might not apply. Equating duration of therapy to cost is not necessarily straightforward. In this trial, a strategy with VUS1 actually used more drug (mean 85 days per cure including retreatment) so is very unlikely to be cost-effective, though VUS2 (mean 63d including retreatment) may be in some circumstances, depending on local costs for treatment. Whether such an approach is viable will depend on the local health system context. A paragraph in this respect is added to the discussion. We have not concluded long term follow up analysis, although agree this will be of some interest in the future.

**Comment:** In the discussion, the rationale of different treatment duration based on baseline viral load needs to be explained, as compared to viral response to DAAs during therapy. This is because baseline VL is not the only factor that affect treatment response. The viral kinetic response might be a more important factor to predict SVR with VUS.

**Response:** We agree that baseline VL is only one factor affecting treatment response and on treatment kinetics may have a role to play. The design of this study was to explore the potential for stratification based on baseline characteristics which would not require more intensive monitoring of patients. The rule was determined from the mean and standard deviation baseline viral load, and the mean estimated declines, from trials published at the time (mean screening VL ~6.25 log _10_ IU/ml, SD 0.4; mean estimated decline 2.15 log _10_ IU/ml per week). Together these were used to estimate the duration of treatment likely to be needed to reduce levels to ~1 copy in the whole body at end of treatment (<0.0001 IU/ml), including a conservative assumption of a moderate negative correlation between baseline and decline in viral load since no data were available on this parameter.

 As demonstrated in the secondary analysis of this study, on treatment responses may well have a helpful role in therapy. Additional comments have been added in relation to these issues in the discussion.

# Peer review: Reviewer response for version 1

**DOI:** 10.21956/wellcomeopenres.18288.r43633

**Published:** 2021-05-11

**Authors:** Kittiyod Poovorawan

**Affiliations:** 1 Mahidol-Oxford Tropical Medicine Research Unit, Mahidol University, Bangkok, Thailand; 2 Department of Clinical Tropical Medicine, Faculty of Tropical Medicine, Mahidol University, Bangkok, Thailand

Reviewer's report: The manuscript entitled " Strategic treatment optimization for HCV (STOPHCV1): a randomised controlled trial of ultrashort duration therapy for chronic hepatitis C " This study aim to evaluate strategies for short-course treatment among chronic hepatitis patients genotype 1a/1b/4 with mild liver disease. This randomised clinical trial is well designed and provides informative results. The study reveals a good data about HCV treatment especially shorter duration according to baseline HCV RNA level. The article is well written and provides beneficial information.

 Reviewer’s Comments

 Minor comments

- According to the study data, the main result seem to reveal that shorter duration of treatment decrease SVR, and in developing country among those treatment failure, re-treatment is complicated and seem to be less cost effective. This would be good if authors provide this issue in the discussion part.

Is the work clearly and accurately presented and does it cite the current literature?

Yes

If applicable, is the statistical analysis and its interpretation appropriate?

Yes

Are all the source data underlying the results available to ensure full reproducibility?

Yes

Is the study design appropriate and is the work technically sound?

Yes

Are the conclusions drawn adequately supported by the results?

Yes

Are sufficient details of methods and analysis provided to allow replication by others?

Yes

Reviewer Expertise:

Gastroenterologist, Viral hepatitis.

I confirm that I have read this submission and believe that I have an appropriate level of expertise to confirm that it is of an acceptable scientific standard.

# Response

**Published:** 2021-07-14

**Authors:** Graham Cooke

**Affiliations:** Imperial College London, UK

**Reviewer 1**

**Comment:** According to the study data, the main result seem to reveal that shorter duration of treatment decrease SVR, and in developing country among those treatment failure, re-treatment is complicated and seem to be less cost effective. This would be good if authors provide this issue in the discussion part

**Response:** The issue of the complexity and cost of retreatment will differ between health systems, as will the relative cost-effectiveness of retreatment. We recognise in the discussion that a universal application of short course treatment, even if relatively cost-effective, would generally be impractical. The intention of the study was to allowing a better understanding of which patients could be selected for shortened course therapy. The cost-effectiveness of any approach requires more detailed analysis in different settings. We have previously published work on the cost-effectiveness of an unstratified short course therapy in an NHS setting (Fawsitt *et al.* 10.1016/j.jval.2018.12.011) and will publish further analysis informed by the STOPHCV1 trial results. A paragraph has been added to the discussion to acknowledge these issues.
